# Graphene oxide classification and standardization

**DOI:** 10.1038/s41598-023-33350-5

**Published:** 2023-04-13

**Authors:** Katarzyna Z. Donato, Hui Li Tan, Valeria S. Marangoni, Marcos V. S. Martins, Pei Rou Ng, Mariana C. F. Costa, Purvi Jain, Sarah J. Lee, Gavin K. W. Koon, Ricardo K. Donato, A. H. Castro Neto

**Affiliations:** 1 Centre for Advanced 2D Materials, National University of Singapore, Singapore, 117546 Singapore; 2 Department of Materials Science and Engineering, National University of Singapore, Singapore, 117575 Singapore; 3 Institute for Functional Intelligent Materials, National University of Singapore, Singapore, 117544 Singapore; 4 Present Address: Ilum School of Science, Brazilian Center for Research in Energy and Materials (CNPEM), Campinas, 13083-970 Brazil; 5 Present Address: Department of Physics and Astronomy, The University of Manchester, Manchester, M13 9PL UK; 6 Present Address: National Graphene Institute, The University of Manchester, Manchester, M13 9PL UK; 7 Present Address: Nanyang Technological University, Singapore, 639798 Singapore

**Keywords:** Materials chemistry, Materials science, Graphene

## Abstract

There is a need to classify and standardize graphene-related materials giving the growing use of this materials industrially. One of the most used and more difficult to classify is graphene oxide (GO). Inconsistent definitions of GO, closely relating it to graphene, are found in the literature and industrial brochures. Hence, although they have very different physicochemical properties and industrial applications, commonly used classifications of graphene and GO definitions are not substantial. Consequently, the lack of regulation and standardization create trust issues among sellers and buyers that impede industrial development and progress. With that in mind, this study offers a critical assessment of 34 commercially available GOs, characterized using a systematic and reliable protocol for accessing their quality. We establish correlations between GO physicochemical properties and its applications leading to rationale for its classification.

## Introduction

Graphene oxide (GO) is a member of a family of two-dimensional (2D) materials, derived from the oxidation of 2D graphitic structures (sp^2^ to sp^3^ carbon conversion). As any other 2D material, in powder form GO presents a statistical nature in terms of its thickness and lateral size distribution. However, GO is an amorphous and non-stoichiometric 2D material, bearing a blend of different functional oxygen groups^1^. In fact, there is no consensus on how to represent the structural model for GO. Hence, important structural details are often neglected, including metallic impurities, functional groups of other heteroatoms, carbon vacancies and radicals, and C–H bonds, which directly depend on the oxidation method used^2^.

There is a plethora of methods available to obtain GO, including the chemical, electrochemical and microbial oxidation of a variety of carbon-based materials^3–6^. The most common results found in the literature involve GO obtained via chemical oxidation of graphite or, most recently, the direct fast oxidation of graphene^7^. However, when considering commercially available GO, it narrows down to the almost exclusive use of chemically oxidized graphite.

Graphite oxidation dates back to 1859 when Benjamin Brodie developed the so-called graphic acid^8^. Later many different approaches were developed aiming to improve Brodie’s method^2,9^. These modifications always involve the addition of a new reactant that leaves its physical footprint in terms of residues and defects that consequently affects GO’s applicability^10^. For instance, the methods developed by Staudenmaier^11^ and Hofmann^12^ methods optimize Brodie’s approach with the use of potassium chlorate (KClO_3_) and nitric acid (HNO_3_). On the other hand, the vastly used Hummers method^13^ applies a mixture of sulfuric acid, sodium nitrate, and potassium permanganate (KMnO_4_) to graphite, yielding materials with very different electrochemical properties than the ones obtained by the other aforementioned methods^14^. This issue is further aggravated by the fact that graphene and GO, in low dose, are not toxic per se, but their cytotoxicity emerges from defects and contaminants^15^.

In this article we survey the overall reliability and consistency of commercially available GO from producers around the world. The closest standardized characterization protocol available for GO is the ISO/TS 21356-1:2021^16^ that was developed for graphene and, with few exceptions^17–19^, no adaptation was tried for GO. Moreover, most of the literature focuses on identifying specific phenomena for small sets of samples. Therefore, we propose a guideline for sample preparation and characterization, including a flowchart that combines up-to-date laboratory common practices for GO characterization (Supplementary Information, SI, Figure S1). Detailed experimental descriptions for individual analyses are extensively discussed in individual sections, focusing on the challenges related to a large number of heterogeneous samples.

A total of 34 different commercially available GO samples were acquired from 25 companies located in 11 countries, 23 of them were obtained as powders and 11 as dispersions in water (5 of these from the same producer as a powder sample), with a price range varying from USD$ 0.40 to USD$ 2300 per gram. This colossal price variation and the lack of information about the synthetic method adopted by most GO producers are exemplary results of the lack of standardization. A blind analysis process was defined, where all the analysts performed the characterizations without knowing any information about the samples. Furthermore, 2 of the 34 products were acquired under a different name than GO, *i.e.*, hydroxyl- and carboxy-functionalized graphene, to act as decoys for the analysts. This was made to evaluate if these samples clearly behave as outliers or stay undetectable among the GO samples, helping to define how broad the GO term can be used, and how similar GO and modified graphene can be within the current product standards.

### Flakes’ lateral size and thickness

Concomitantly with any other member of the 2D materials family, the GO dimensions imply dramatic effects on its properties, to such an extent that it can define the formation of liquid crystal phases in water^20^. Consequently, variations in the dimensions of GO flakes yield different products for different applications, especially in areas such as polymer composites where interphase effects are critical^21,22^.

**Figure 1:**
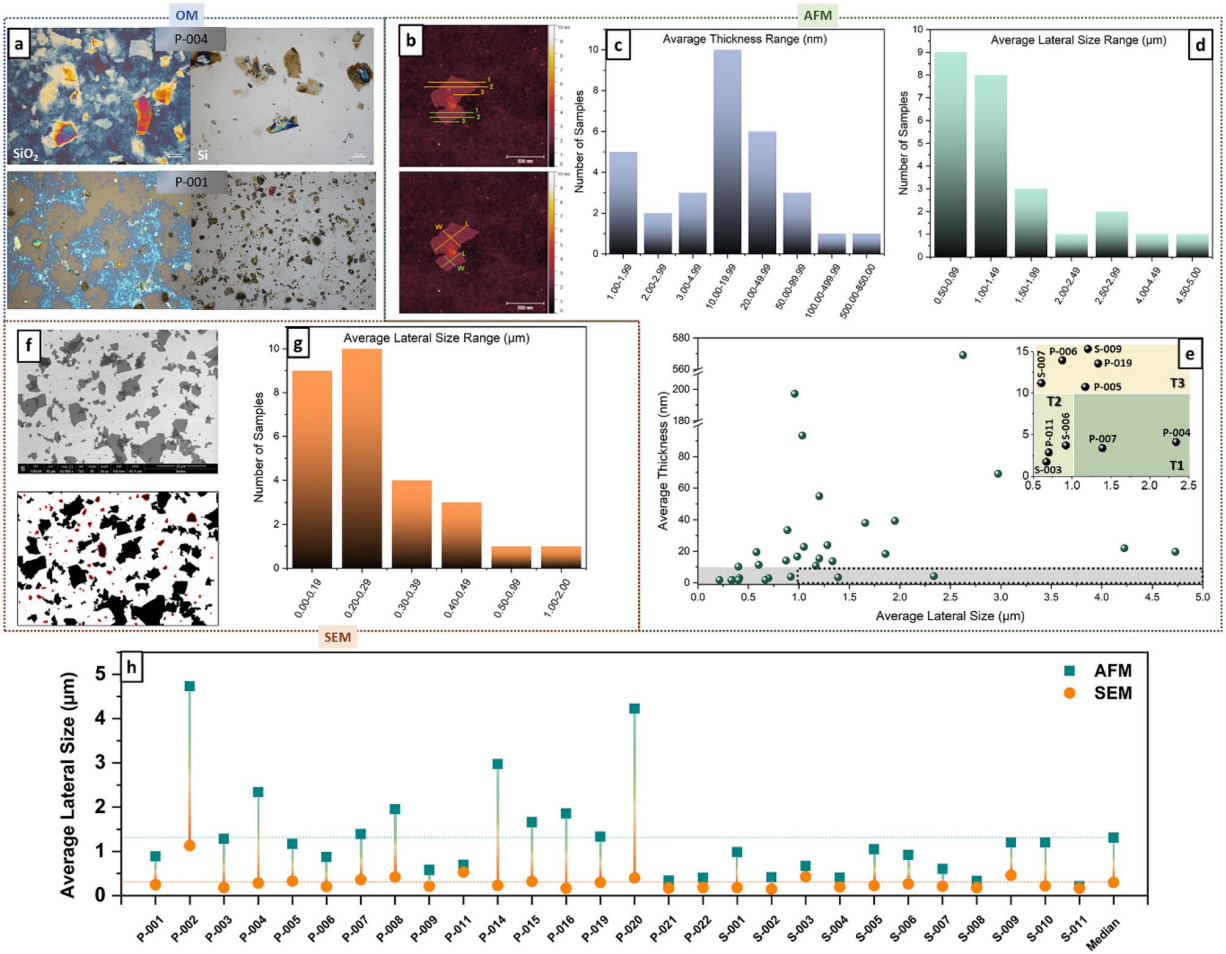
Thickness and lateral size determination. (**a**) Examples of OM images of samples characterized using Si/SiO_2_ or Si substrates. (**b**) Examples of thickness and lateral size collection by AFM, followed by the distributions of average (**c**) thickness and (**d**) lateral size, and (**e**) correlation between thickness and lateral size for each sample with the inset representing 3 different Tiers (T1 = *h* ≤ 10 nm and *l* ≥ 1 μm; T2 = *h* ≤ 10 nm and 1 μm > *l* ≥ 0.5 μm; T3 = 15 nm ≥ *h* > 10 nm and *l* ≥ 0.5 μm). (**f**) Example of SEM image for lateral size collection, highlighting the smaller often neglected flake fragments, followed by (**g**) the lateral size distribution using a software interface. (**h**) Finally, the difference between the average lateral size obtained from AFM (manually) and SEM (software).

Optical microscopy (OM) is the simplest method to estimate the lateral size of GO’s flakes. However, it can only be used to observe sample appearance prior to more quantitative characterizations, as defined by the ISO norm related to graphene^16^. Although this approach is often found in the GO literature, we observe a large discrepancy among samples and their optical definition when using the ISO-recommended substrate composition (Si/SiO_2_) for non-purified commercial GOs. Samples with large flakes and fewer impurities are clearly observed with OM, whereas smaller and more contaminated flakes are barely visible due to artifacts created by the interaction between the Si/SiO_2_ substrate and organic contaminants. This can be partially resolved by using Si substrates, although losing the qualitative information of flake thickness^23^ (Fig. 1a). For this reason, all samples were analyzed using both Si and Si/SiO_2_ substrates, and the one presenting the most defined flake morphology and distribution was used for further characterizations (Figure S2).

The flake thickness distribution of the samples was assessed by AFM, considering the height profile (in triplicate) of at least 30 individual flakes per GO sample (Fig. 1b and details in the SI, Sect.  4, Figure S3). In total, 31 of the originally acquired samples could be analyzed by AFM and 3 of them were above the thickness range allowed for the measurement. From the measured samples, 20 presented an average thickness < 20 nm, among which only 10 were < 10 nm (Fig. 1c). All the other samples were deliberately treated as graphite oxide or partially oxidized graphite. Moreover, considerable differences were also observed between GOs acquired as powders or dispersions. The segregated values of average flake thickness, as well as the D_50_ and D_90_, are shown in SI (Figure S4). Interestingly, most of the samples acquired as suspensions were thin (> 50% < 10 nm), while < 20% of samples acquired as powders were < 10 nm thick. Furthermore, the D_50_ and D_90_ values of powder samples are very discrepant, whereas they are much more consistent in suspension samples, indicating a narrower thickness distribution. These differences are also evident when comparing powder and dispersion samples acquired from the same producer (e.g. P-008 and S-009), showing they are arising from a processual step and not necessarily from lack of quality control. This may not come as a surprise since drying processes may lead to strong physical interactions, reactions among functional groups, or even the reduction of the oxygenated groups of GO, all leading to irreversible sheet restacking^24,25^.

The flakes’ average lateral size was also measured by AFM, according to the procedure described in the SI, and illustrated in Figs. 1b and S3. All acquired samples present an average lateral size < 5 μm, and the vast majority were < 2 μm (Fig. 1d). This was quite unexpected since an appealing characteristic of (chemically oxidized) GO is exactly the reduced sheet ripping, implying a larger lateral size in comparison to methods that demand some kind of mechanical exfoliation^26^.

The unique properties of the 2D materials are intrinsically related to their anisotropy, thus, comparing the relationship between the thickness and lateral size of each sample made this situation even more alarming. About 80% of samples with a thickness ≤ 10 nm have an average lateral size below 1 μm (Fig. 1e). Indeed, we have arbitrarily set thresholds for a maximum thickness or height (*h*) and minimum lateral size (*l*) as 3 different Tiers (Fig. 1e, inset), roughly considering values that are acceptable for different application. Although the limits applied were not very stringent, only 2 samples passed the minimum parameters for T1 (dotted area in Fig. 1e), while 3 samples qualified for T2 and another 5 to T3 (Fig. 1e, inset). Moreover, the use of a large number of flakes per sample alleviates the statistical biases in manual calculations, but it does not avoid cognitive biases related to choosing large flakes rather than small ones (saliency bias). For this reason, the GO lateral size was also characterized with SEM and the data treatment was performed with a software interface, avoiding manual counting and reducing human-related errors (details in the SI, Sect.  5 and Figure S5). Since this method takes into consideration also ripped off parts and debris (above 100 nm) from the large and most integral flakes (Fig. 1f), it gives a more realistic depiction of the overall GO content purchased as a product (especially when considering the large-scale use in industry). Although we recognise this method has limitation, we believe it could largely benefit from current technological improvements, e.g., by adapting an A.I. system to recognize how a GO flake looks like.

The size distribution obtained by this method is expectedly smaller than by AFM, revealing an average lateral size of > 50% of the samples is < 300 nm. This shows that a large number of flakes is neglected during manual counting and, although the small and fragmented flakes represent the debris of the preparation process, they are a considerable part of the total composition and can cause meaningful effects in different applications. Figure 1h shows the difference between average lateral sizes obtained by AFM and SEM for each sample, where the size of the line between the values is proportional to the content of neglected smaller flakes and fragments.

### Degree of oxidation, defectiveness, and contamination

**Figure 2:**
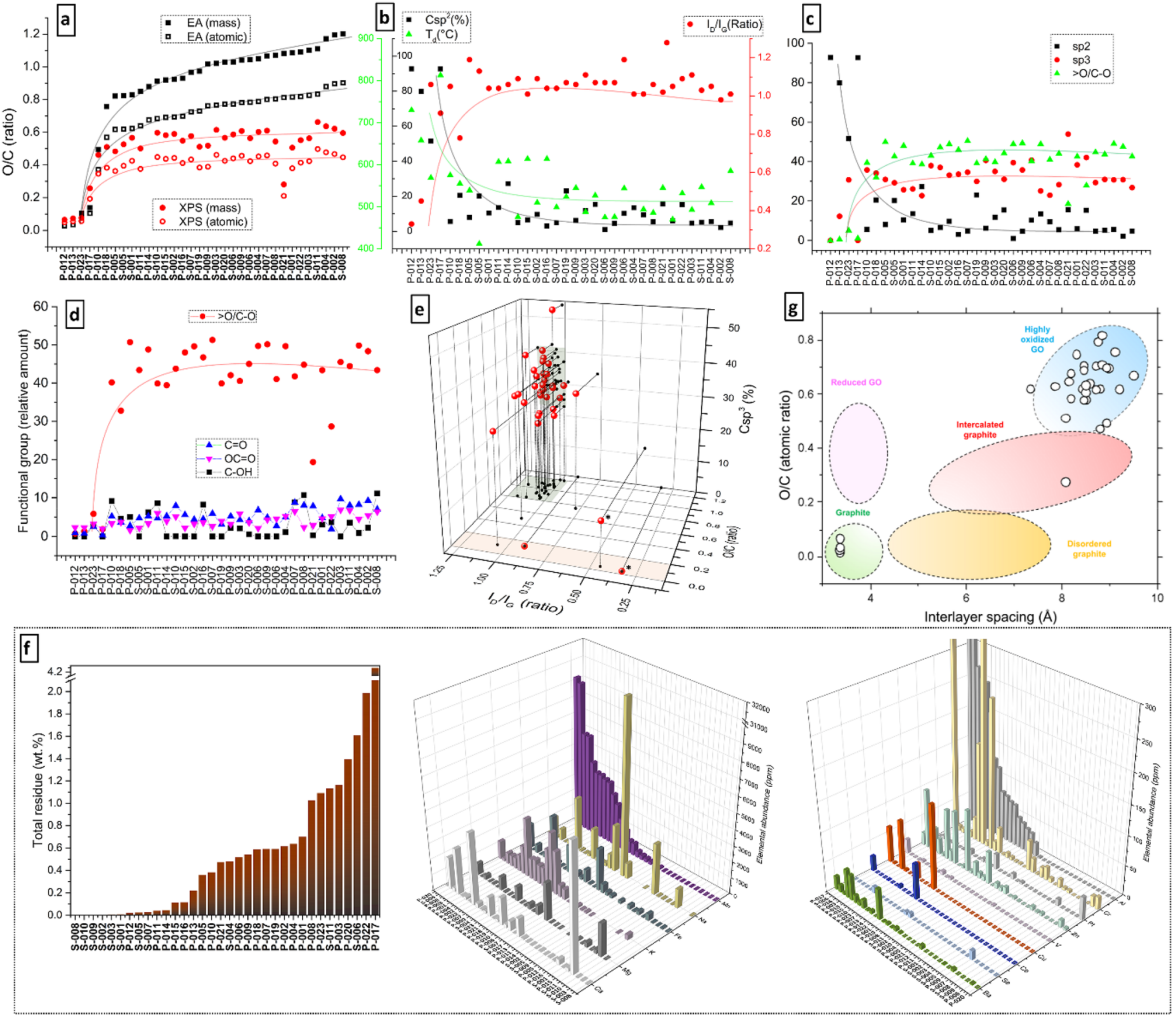
Degree of oxidation and defectiveness of GOs. Comparison of both mass and atomic O/C ratios from XPS and EA (**a**). Correlation between C sp^2^ (XPS), I_D_/I_G_ (Raman), and T_d_ (TGA) (**b**). C sp^2^ consumption related to C sp^3^ and epoxy functionalities (> O/C-O) (**c**). Relative amounts of > O/C-O, C=O, OC=O, and C–OH. Correlation between I_D_/I_G_, degree of oxidation (O/C, EA), and amount of C sp^3^ (XPS) (**e**). Total residue percentage together with Ca, Mg, K, Fe, Na, Mn, Ba, Se, Ca, Cu, V, Zn, Pt, Cr, and Al trace element abundance (ICP-OES) (**f**). Correlation between interlayer spacing (XRD) and O/C ratio (EA) for all the samples, and their comparison with other carbonaceous materials (**g**).

Besides its GO dimensions, another critical issue-defining GO’s quality is the fact it is an oxide. Thus, the degree of oxidation and the types of oxygen-bearing groups it contains define its range applicability^27^. The most commonly used analytical methods to define the degree of oxidation and functionalities in GO are elemental analysis and XPS. Due to its larger availability and smaller amounts of sample required for characterization, XPS is often used to cover both oxidation and functional group determinations^28^. Moreover, for similar GO samples presenting variations in the oxygen content, the degree of oxidation can be precisely determined using a combination of XPS and computational methods^1^, and correction methods are available to mitigate the effect of extraneous oxygen contamination^28^. However, since our target samples are prone to heterogeneity and XPS is a surface-targeted analysis, we decided to interpolate the oxygen/carbon ratios (O/C) obtained by both XPS (SI, Sect. 6, Table S1) and elemental analysis (EA, SI Sect. 7, Table S2). For easier visualization of the correlations, in Fig. 2, the GO samples were ordered with ascending O/C values (as obtained by EA). Indeed, a large discrepancy between XPS and EA builds up with increasing oxygen content (Fig. 2a). The O/C elemental ratios obtained by both methods are comparable only in the 4 GO samples with low O content. For the rest of the samples, XPS-based O/C values are lower by ~ 0.2–0.3 in comparison to EA, including extreme cases where the values differ by > 50% (Fig. 2a). Since EA is a bulk-based analysis and uses large amounts of sample, we adopt its O/C and C/O values as a reference, while XPS is used only for functional group quantification.

Important correlations are obtained by interpolating XPS, Raman spectroscopy, and TGA (Fig. 2b, details in SI, Sects. 6, 8, and 9, respectively). Since the samples are ordered with ascending O/C values, the increased degradation of the crystalline basal plane can be witnessed by the decreasing amount of C sp^2^ (by XPS) and increased number of defects (I_D_/I_G_ ratio by Raman), leading to decreased thermal stability (by TGA). The C sp^2^ consumption also correlates directly with the increase in C sp^3^ and the formation of epoxy functionalities (> O/C–O), all obtained by XPS (Fig. 2c). We chose > O/C-O as a reference oxidation group because at higher degrees of oxidation it becomes the dominant functionality. With only 2 exceptions, all GOs with O/C > 0.5 form a plateau at ~ 45% > O/C-O in their composition, as witnessed in Fig. 2d. Although on a much smaller scale, C–OH functionalities also vary largely among GO samples (Fig. 2d), which is a clear fingerprint of the presence of water during the chemical oxidation process^29,30^. A clear correlation emerges when we interpolate the information about the GOs’ defects by Raman (I_D_/I_G_), their degree of oxidation by EA (O/C), and their amount of sp^3^ carbon by XPS (C sp^3^) (Fig. 2e). Relatively small distribution regions could be attributed to these values, where 27 out of 34 GO samples presented 0.9 ≤ I_D_/I_G_ ≤ 1.2, 0.8 ≤ O/C ≤ 1.2 and 25% ≤ % C sp^3^ ≤ 42%. Among the 7 outliers are the 2 decoy GO samples we introduced, presenting a clear differentiation from the highly defective and low oxidation “GOs”.

During the oxidation process, O-related functionalities are added among the graphite layers and increase the interlayer spacing (*d*), which can be followed by XRD. Ideally, the characteristic diffraction (002) peak of graphite (2θ ~ 26.3°, *d* ~ 3.4 Å) should completely disappear, and all the remaining diffraction structure at this (°) is residual unoxidized graphite^31^. At least 5 out of 34 samples analyzed present intense residual (002) peaks, and several others presented it in lower intensities (SI, Sect. 10, Figure S13). Moreover, the (001) peak is formed as a result of oxidation and *d* varies related to the type of modification introduced to the basal plane of graphite. The characteristic GO (001) peak in 2θ = 9.3–12.1°, corresponding to the *d* = 9.5–7.3 Å, was observed for 30 out of 34 samples (Figure S13). Although it is known^32^ that larger groups and/or more oxygen produce a larger *d*, more variables seem to be at play as this pattern did not emerge for our set of samples. Both FTIR and ATR-FTIR were performed to supplement the XPS information about the functional groups, but the overall heterogeneity of the samples only allowed for qualitative functional group identification (SI, Sect. 11). Importantly, we detected fingerprint bands for sulfate/sulfonate groups that confirm that part of the sulfur residue detected by EA is not only adsorbed on the GOs but covalently bonded (SI, Table S6). On the other hand, we correlate the interlayer spacing and O/C ratios, indicating the carbonaceous group within which the analyzed materials can be associated to. Figure 2g displays this correlation to all tested materials and compares them to values found in the literature for graphite, reduced GO, disordered graphite, intercalated graphite, and highly oxidized GO^31,33,34^. We must highlight that even though these values are estimations without discounting eventual water (or other residual solvents) contributions^35^, the XRD peak segregations were defined enough for interpretation after the sample drying process (SI, Figure S13). Most of the samples fit into the highly oxidized GO region, however, four samples present characteristics of graphite, and one sample resembled more intercalated graphite. Curiously, only our 2 decoy samples were expected to appear in the graphite region (due to the expected low oxidation), however, another 2 samples are also displayed there, and the 4 samples are indistinguishable. The same 4 GOs presented very weak and noisy FTIR spectra and did not display one or more of the GO’s fingerprint vibrational bands (details in the SI, Sect. 11, Figure S14, and Tables S6 and S7). In fact, the decoy samples are only clearly distinguishable when Raman spectroscopy is included to the analytical protocol (Fig. 2e).

Finally, we also investigate the content of residual metallic impurities present in the commercial GOs, using ICP-OES (details in SI, Sect. 12, Table S8). There are two main sources of the metallic residues in GO, the graphite used for oxidation and the major components of the reactants used in the different synthetic steps. However, indirect contamination with extraneous metals coming as trace residues of reactants have also been reported^10^. Consequently, purification steps are essential for the GO syntheses due to their large chemical footprint, but they considerably increase the production cost of GO. Thus, impurities become a point of concern when acquiring GO from a seller. In Fig. 2f, we summarize the total amount (by weight), the major (present in thousands of ppm) and the minor (present in hundreds of ppm) metallic impurities present in the commercial GOs. They were organized in ascending order of the major component of the figure for better visualization. Astonishingly, 8 of the samples presented > 1wt.% of metallic residues, including an extreme case with > 4wt%. The majority of the largely contaminated GOs are powder samples, whilst, with 3 exceptions (S-004, S-006 and S-011), dispersion samples presented orders of magnitude less impurities. The major contaminants observed are Mn and Na, followed by K, Mg, Ca and Fe, present in thousands of ppm. These contaminants are easily traceable since they are part of reaction components and/or cations largely present in water^36^, tracing it back to the water in the washing process. However, Al and Cr were also present in surprisingly large amounts in some GOs (hundreds of ppm), while Pt, Zn, V, Cu, Co, Se and Ba were lesser (but still in tens of ppm in some samples).

### Concentration and water-stability

Among the most unexpected conclusions we have reached from this study is that the unreliability of commercially available GO starts from the products’ contents. The GOs acquired as powders were tested for their dispersibility and apparent stability in water and compared to the ones acquired already as dispersions (SI, Sect. 13 and Figure S16). From the 23 powder samples, 9 of them presented very poor stability in water, not forming homogeneous suspensions and precipitating shortly after sonication. Moreover, among the initially stable powder samples, 70% of them precipitated in < 30 days. The GOs acquired as dispersions generally show better water stability, keeping stable or forming easily re-dispersible phase separations even after 30 days of storing (more details below).

**Figure 3:**
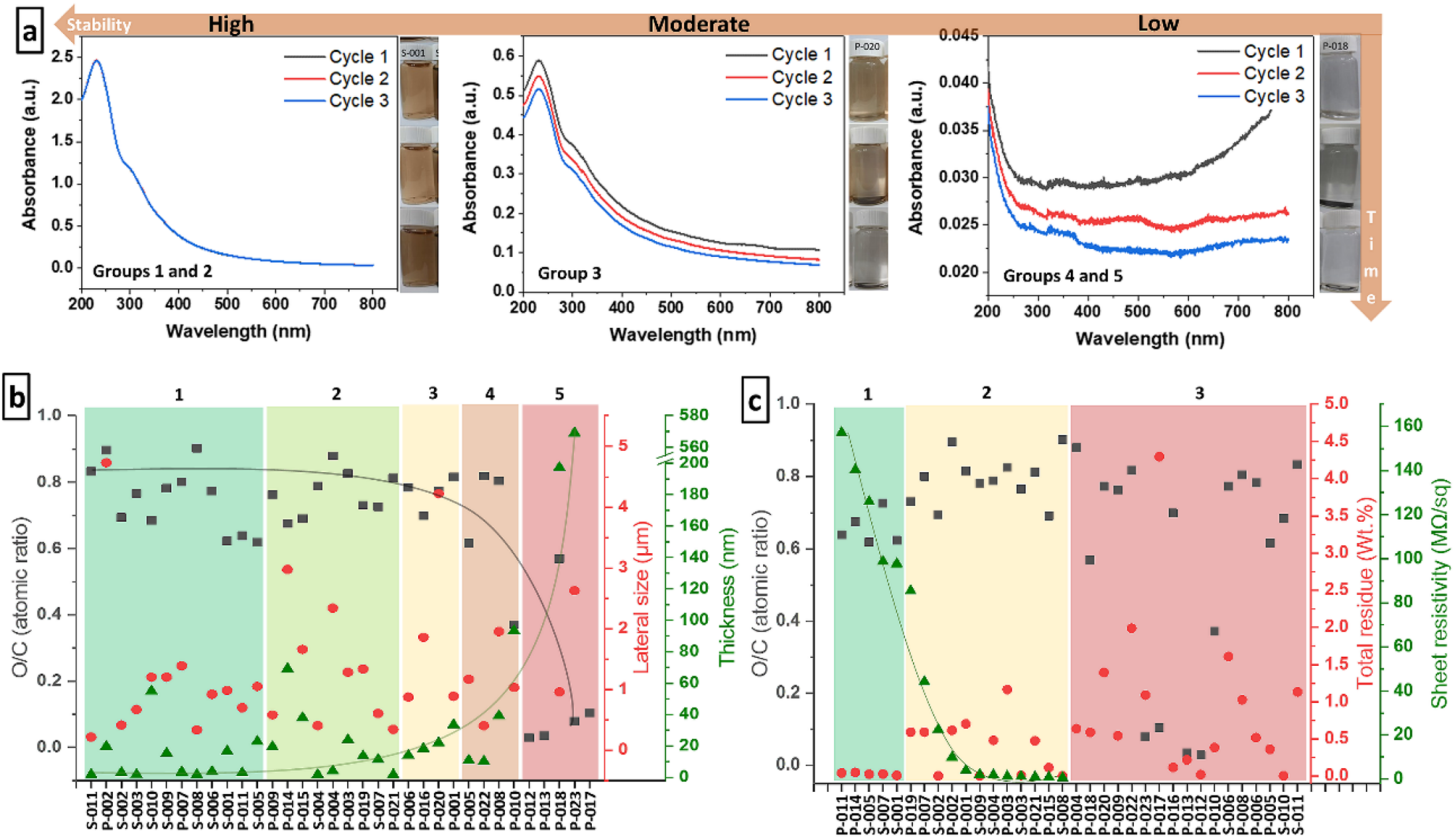
Samples with different levels of stability, with image insets showing examples of water dispersions just after sonication (top), after 24 h (middle), and after 30 days of storing (bottom) (**a**). Correlation between O/C ratio, lateral size, flake thickness, and the GOs’ water stability, grouping the sample by stability from 1 to 5 (details in SI, Sect. 15) (**b**). Correlation between O/C ratio, total residue, and sheet resistivity, grouping the samples by film formation ability where group 1 gather samples that form films with high resistivity, group 2 form films with decreasing resistivity and group 3 does not form films (**c**).

On the other hand, when we performed the gravimetric analysis of the solid content in the acquired GO suspensions, unveiling their absolute concentrations in water, it revealed a large mismatch between the labeled concentration and the real concentration (SI, Sect. 14 and Table S9). From the 11 dispersion samples, 4 presented a standard deviation >  ± 0.5 mg/mL, and 1 sample presented a concentration 5 × lower than the value described in the product label. This is particularly concerning because many researchers rely on UV–Vis analysis of GO to establish its concentration. However, such a heterogeneous group of samples is not comparable via this technique. Since the optical absorption of GO is dominated by the π–π* plasmon that is dependent on the linking chromophore units (e.g., C=C, C=O, and C–O bonds), variations on those units will strongly affect the concentration determination^37^. This can be clearly evidenced by the large variation in absorbance among the different commercial GOs when characterized under the same concentration (0.05 mg/mL), including 9 of them that do not even present a typical GO UV–Vis absorption curve (SI, Sect. 15 and Figure S17). These differences in content and type of O-groups also lead to large differences in water stability as previously described (SI, Sect. 13, Figure S15). The stability in water, as opposed to the dispersion concentration, can be reliably determined by UV–Vis using a simple method we propose (Fig. 3a, and details in SI, Sect. 15). Briefly, we classify the GOs into 5 different solution stability groups, presenting also different features that can be observed by UV–Vis, which accurately match with the apparent water stability (Fig. 3b, and details in SI, Sect. 12). Indeed, only 21 of the 34 commercial GOs presented good stability in water, which is a highly regarded property that should be expected from any GO product.

There is a complex interplay between flake sizes and oxidation controlling water stability, thus, the correlation between O/C ratio, lateral size, and flake thickness of the GOs sets a good guideline to understand their water stability. Not surprisingly, the water stability of the commercial GOs decreases with increasing average flake thickness and decreasing O/C (Fig. 3b). However, interestingly, the influence of flake lateral sizes on the GOs’ water stability seems to be little to none, as observed by the scattered results when the samples are organized in order of their group of stability (Fig. 3b). In fact, the most unstable samples as per UV–Vis determination (Group 5, SI, Sect. 15) are exactly the samples with extremely low O/C ratio and/or extremely thick flakes, including the 2 (non-GO) decoy samples. There is also a major influence of the metallic residue of the samples which will be detailed in the film formation discussion.

Although surface charge density usually confers a large influence on the stability of particles in solution^31^, we could not observe any major influence of it in this set of samples as the difference in GO stability was not directly assessable by characterizing their electrophoretic properties. The dispersion of commercial GOs presented a large difference in pH (pH 4.4–8.6 at 0.05 mg/mL, SI, Sect. 16 and Table S10). This is an indicator of large differences in synthesis protocols adopted and, consequently, residual additives, which we clearly observed (Fig. 2f). In fact, for many of the samples the amount of contamination is so high that it seems to be the dominant effect on the pH variation and instability. For a fair comparison among the samples, zeta potential (ζ) analysis was performed both in the original pH and after having the pH adjusted to 6–7, to ensure a stable pH range for GO^38^. The very unstable samples are not measured, as they do not present stable or reliable ζ values. All the other GOs previously presenting from moderate to high stability displayed ζ < -30 mV in the original pH and ζ < -40 mV when their pH was adjusted to pH 6–7, indicating a high enough surface charge density to guarantee stable particle dispersions. More details are presented in the SI (Sect. 16, Table S10 and Figure S18).

### Film formation and electronic properties

The film formation properties are among the most desired properties of GO making it applicable in areas as diverse as programable membranes for ion segregation^39^, water purification and desalination^40^, biomedical sensors^41^, and mechanically reinforced nanocomposites^42^. For this reason, following the study on the water stability of the commercial GO samples, we test their film formation properties. Moreover, since GO is well-known as an insulator and this condition is dependent on its degree of oxidation^43^, we characterize the sheet resistivity of the films formed. Due to the heterogeneity that is anticipated for the samples, and the knowledge that impurities and oxidative debris tend to collapse the GO film structure^44^, we facilitate the film formation by applying a solvent mixture/isopycnic centrifugation-based method^45^. This is made to increase the chances of film formation, improve the quality of formed GO films, and, consequently, increase the accuracy of measurements (details in SI, Sect. 17).

Surprisingly, only half of the GO samples could form a film or a continuous structure that was stable enough to be characterized. In fact, only about 6 out of the 34 samples were able to form continuous and homogeneous films, all derived from commercial GO dispersions (SI, Sect. 17, Figure S19). Moreover, except for one GO belonging to stability group 3, all GO samples that can form films belong to dispersion stability groups 1 and 2 (Fig. 3b), confirming an obvious correlation between GO stability and film quality.

Concerning the electrical resistivity measurements, the results range from 0.85 MΩ/sq to 157.12 MΩ/sq, more than 2 orders of magnitude difference. These results are a direct effect of the large disparities unveiled for the structure (Fig. 1) and composition (Fig. 2), and affecting the stability (Fig. 3) across the GO samples. In fact, only samples with high degree of oxidation and relatively low contamination presented resistivities above 100 MΩ/sq (Fig. 3c, region 1). A sharp drop in resistivity is observed for most samples with increased contamination (but values still ≥ 1 wt.%), as demonstrated in Fig. 3c, region 2. All the remaining sample presenting high contamination and/or low degree of oxidation do not form films and, thus, have no value computed (Fig. 3c, region 3).

## Summary and outlook

After our extensive evaluation of the quality of GOs worldwide, we concluded that only 4 out of the 34 samples analyzed display an acceptable performance within our protocol and deliver approximately what they display on the label or brochure as the content of their commercial product. Similarly to previously reported for graphene^46^, residual contamination from processes and prime materials, and flake thickness were major issues, but in a much larger scale due to the heavy chemical footprint of the GO production process (about half of the samples presented > 5000 ppm in metallic residues). However, inconsistencies in lateral size and degree of oxidation were also vast, to an extent that different analytical methods yielded different results due to the heterogeneity.

Finally, we would like to highlight that this critical assessment of the quality of commercial GOs should be taken as an eye-opener, and was designed with the intention to promote and suggest directions to speed up the standardization of GO as a product. Understanding that the fields of GO application are vast, and considering the many steps involved in GO’s preparation, after this extensive and laborious analytical exercise we prospect that an all-in-one quality control protocol is most likely unattainable. However, we could establish 3 main cores of evaluation that may guide the adaptation of these protocols to the specific needs. The first 2 are structurally focused and consist in the dimensional (Fig. 1) and compositional (Fig. 2) determination of the GOs, while the third one correlates the first 2 with their dispersion stability and film formation (Fig. 3). Altogether, we anticipate that well-regulated and standardized application-based grades are the way-to-go for GO, since they can accommodate the limitations and tolerances allowed by individual applications.

### Experimental section

Materials: the total of 34 GOs were purchased from 25 commercial sources. Samples were obtained either in powder form (23) or water dispersion (11) and were analyzed as received following the protocols that are described in detail in the SI.

Characterizations: Sample preparations and characterizations are described in detail in the SI.

## Supplementary Information

Supplementary Information.

## Data Availability

The raw/processed data required to reproduce these findings are available upon reasonable request to the corresponding authors (donato@nus.edu.sg or c2dhead@nus.edu.sg).

## References

[1] Carvalho A (2021). The degree of oxidation of graphene oxide. Nanomaterials.

[2] Brisebois PP, Siaj M (2020). Harvesting graphene oxide-years 1859 to 2019: A review of its structure, synthesis, properties and exfoliation. J. Mater. Chem. C.

[3] Nishina Y, Eigler S (2020). Chemical and electrochemical synthesis of graphene oxide – a generalized view. Nanoscale.

[4] Yang H (2019). Fungal transformation of graphene by white rot fungus Phanerochaete chrysosporium. Chemosphere.

[5] Liu L (2015). Oxidation and degradation of graphitic materials by naphthalene-degrading bacteria. Nanoscale.

[6] Lu L (2015). Graphene oxide and H2 production from bioelectrochemical graphite oxidation. Sci. Rep..

[7] Costa MCFF (2021). Accelerated synthesis of graphene oxide from graphene. Nanomaterials.

[8] Brodie BCXIII (1859). On the atomic weight of graphite. Philos. Trans. R. Soc. London.

[9] Chen D, Feng H, Li J (2012). Graphene oxide: Preparation, functionalization, and electrochemical applications. Chem. Rev..

[10] Wong CHA (2014). Synthetic routes contaminate graphene materials with a whole spectrum of unanticipated metallic elements. Proc. Natl. Acad. Sci..

[11] Staudenmaier L (1898). Verfahren zur Darstellung der Graphitsäure. Berichte der Dtsch. Chem. Gesellschaft.

[12] Hofmann U, König E (1937). Untersuchungen über Graphitoxyd. Zeitschrift für Anorg. und Allg. Chemie.

[13] Hummers WS, Offeman RE (1958). Preparation of graphitic oxide. J. Am. Chem. Soc..

[14] Poh HL (2012). Graphenes prepared by Staudenmaier, Hofmann and Hummers methods with consequent thermal exfoliation exhibit very different electrochemical properties. Nanoscale.

[15] Malhotra R (2022). Cytotoxicity survey of commercial graphene materials from worldwide. npj 2D Mater. Appl..

[16] ISO/TS 21356–1: Nanotechnologies — Structural characterization of graphene — Part 1: Graphene from powders and dispersions. vol. 2011 (2011).

[17] de Oliveira Cremonezzi JM, Ribeiro H, Andrade RJE, Fechine GJM (2022). Characterization strategy for graphene oxide and molybdenum disulfide: Proceedings based on the ISO/TS 21356–1: 2021 standard. FlatChem.

[18] Albers PW (2022). The characterisation of commercial 2D carbons: Graphene, graphene oxide and reduced graphene oxide. Mater. Adv..

[19] Amadei CA, Arribas P, Vecitis CD (2018). Graphene oxide standardization and classification: Methods to support the leap from lab to industry. Carbon N. Y..

[20] Jalili R (2014). Formation and processability of liquid crystalline dispersions of graphene oxide. Mater. Horiz..

[21] Jiang W (2022). Exploring the size effect of graphene oxide on crystallization kinetics and barrier properties of poly(lactic acid). ACS Omega.

[22] Garcia PS (2021). Tailoring the graphene oxide chemical structure and morphology as a key to polypropylene nanocomposite performance. Polym. Compos..

[23] Jung I, Rhyee JS, Son JY, Ruoff RS, Rhee KY (2012). Colors of graphene and graphene-oxide multilayers on various substrates. Nanotechnology.

[24] Yoon Y (2013). Anti-solvent derived non-stacked reduced graphene oxide for high performance supercapacitors. Adv. Mater..

[25] Dong Y, Li J, Yang X-Y (2022). Reactions between graphene oxide sheets cause irreversible agglomeration. Sci. Bull..

[26] Wang X-Y, Narita A, Müllen K (2018). Precision synthesis versus bulk-scale fabrication of graphenes. Nat. Rev. Chem..

[27] Dideikin AT, Vul’ AY (2019). Graphene oxide and derivatives: The place in graphene family. Front. Phys..

[28] Kovtun A (2019). Accurate chemical analysis of oxygenated graphene-based materials using X-ray photoelectron spectroscopy. Carbon N. Y..

[29] Zhang Q (2021). Roles of water in the formation and preparation of graphene oxide. RSC Adv..

[30] Chen J (2016). Water-enhanced oxidation of graphite to graphene oxide with controlled species of oxygenated groups. Chem. Sci..

[31] Krishnamoorthy K, Veerapandian M, Yun K, Kim SJ (2013). The chemical and structural analysis of graphene oxide with different degrees of oxidation. Carbon N. Y..

[32] Marcano DC (2010). Improved Synthesis of Graphene Oxide. ACS Nano.

[33] Müller-Warmuth, W. & Schöllhorn, R. Progress in Intercalation Research. 536 (1994).

[34] Burchell TD (1999). Carbon Materials for Advanced Technologies.

[35] Talyzin AV (2008). Colossal pressure-induced lattice expansion of graphite oxide in the presence of water. Angew. Chemie Int. Ed..

[36] Wodschow K, Hansen B, Schullehner J, Ersbøll AK (2018). Stability of major geogenic cations in drinking water—An issue of public health importance: A Danish study, 1980–2017. Int. J. Environ. Res. Public Health.

[37] Lai Q, Zhu S, Luo X, Zou M, Huang S (2012). Ultraviolet-visible spectroscopy of graphene oxides. AIP Adv..

[38] Shih C-J, Lin S, Sharma R, Strano MS, Blankschtein D (2012). Understanding the pH-dependent behavior of graphene oxide aqueous solutions: A comparative experimental and molecular dynamics simulation study. Langmuir.

[39] Andreeva DV (2021). Two-dimensional adaptive membranes with programmable water and ionic channels. Nat. Nanotechnol..

[40] Homaeigohar S, Elbahri M (2017). Graphene membranes for water desalination. NPG Asia Mater..

[41] Nguyen EP, Silva CDCC, Merkoçi A (2020). Recent advancement in biomedical applications on the surface of two-dimensional materials: From biosensing to tissue engineering. Nanoscale.

[42] Hegde M (2020). Strong graphene oxide nanocomposites from aqueous hybrid liquid crystals. Nat. Commun..

[43] Eda G, Mattevi C, Yamaguchi H, Kim H, Chhowalla M (2009). Insulator to semimetal transition in graphene oxide. J. Phys. Chem. C.

[44] López-Daz D, Merchán MD, Velázquez MM, Maestro A (2020). Understanding the role of oxidative debris on the structure of graphene oxide films at the air-water interface: A neutron reflectivity study. ACS Appl. Mater. Interfaces.

[45] Cotet LC (2017). Versatile self-assembled graphene oxide membranes obtained under ambient conditions by using a water–ethanol suspension. J. Mater. Chem. A.

[46] Kauling AP (2018). The worldwide graphene flake production. Adv. Mater..

