# Supplementary material for: Graphene oxide classification and standardization
Source: Sci Rep. 2023 Apr 13;13:6064. doi: 10.1038/s41598-023-33350-5 (PMC10102077; doi:10.1038/s41598-023-33350-5)
Supplement: Supplementary file 1 — Supplementary Information. [file 41598_2023_33350_MOESM1_ESM.pdf]

Supplementary Information for: Graphene Oxide classification and standardization

Katarzyna Z. Donato[1], Hui Li Tan[1], Valeria S. Marangoni[1,a], Marcos V. S. Martins[1,b,c], Pei Rou Ng[1,2,3], Mariana C.F. Costa[1,2,3], Purvi Jain[1,d], Sarah J. Lee[1], Gavin K.W. Koon[1], Ricardo K. Donato[1], A.H. Castro Neto[1,2,3*]*

[1] Centre for Advanced 2D Materials, National University of Singapore, 117546, Singapore.
[2] Department of Materials Science and Engineering, National University of Singapore, 117575, Singapore.
[3] Institute for Functional Intelligent Materials, National University of Singapore, 117544, Singapore.

E-mail: donato@nus.edu.sg; c2dhead@nus.edu.sg

Current address:

[a] Ilum School of Science, Brazilian Center for Research in Energy and Materials (CNPEM), 13083-970, Brazil.

[b] Department of Physics and Astronomy, The University of Manchester, M13 9PL, United Kingdom.

[c] National Graphene Institute, The University of Manchester, M13 9PL, United Kingdom.

[d] Nanyang Technological University, 639798, Singapore.

Table of contents

1. Sample Preparation and Characterization Protocol 3

2. Water dispersions 3

3. Optical Microscopy (OM) 4

4. Atomic Force Microscopy (AFM) 5

5. Scanning Electron Microscopy (SEM) 7

6. X-ray photoelectron spectroscopy (XPS) 11

7. Elemental analysis (EA) 14

8. Raman Spectroscopy 15

9. Thermogravimetric analysis (TGA) 19

10. X-ray diffraction measurements (XRD) 22

11. Infrared Spectroscopy 23

12. Inductively Coupled Plasma Optical Emission spectroscopy (ICP-OES) 26

13. Dispersions' apparent stability 29

14. Gravimetric Study 30

15. Ultraviolet–visible spectroscopy (UV-Vis) 31

16. pH and zeta potential measurements 34

17. Preparation of GO films and sheet resistivity measurements 37

18. Individual GO samples profile 40

References 41

1. Sample Preparation and Characterization Protocol

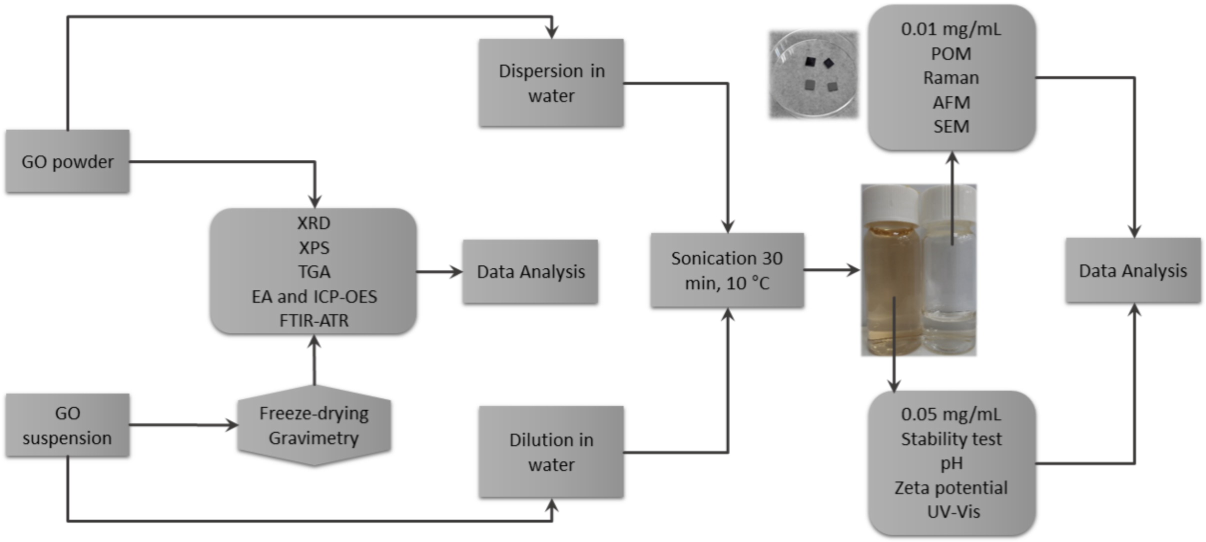

Figure S1. Flowchart for GO characterization.

Figure S1 illustrates the workflow for testing graphene oxide (GO) that is consistent with the scientific literature and the common practice in laboratories worldwide. We have received samples as powders or suspension/slurry. Powder samples were tested as received while suspensions were submitted to the freeze-drying/gravimetric procedure. The lyophilization technique was used to avoid a high-temperature drying and eventual reduction/restacking of GO. All powders were stored in desiccators at least 24 h prior to analyzing. Samples received as powders were named P-xxx while suspensions were named S-xxx. All the samples were characterized using blind analysis.

2. Water dispersions

For powders, 5 mg was resuspended in 100 mL DI water using a volumetric flask while suspensions/slurries were diluted accordingly to their real concentration (see Section 13**)** to obtain a final concentration of 0.05 mg/mL. These suspensions were used for the dispersion stability, pH, and Zeta potential as well as UV-Vis tests. Samples were further diluted to 0.01 mg/mL and drop-casted on Si and Si/SiO_2 wafers for POM, Raman, AFM, and SEM analysis. Si and Si/SiO_2 wafers were dried for a minimum of 24 h in the dissector before the analysis. Directly before suspension analysis or drop-casting, samples were submitted to 30 min

ultrasound at 10 °C (Elmasonic S60H ultrasonic bath, 37 Hz). The time of exposure to sonication was chosen for the minimal flake size disruption, but guarantying a good enough exfoliation of the GOs for a fair comparizon[1].

3. Optical Microscopy (OM)

OM can be used as initial quick and non-destructive quality indicator of GO sample. GO flakes are mostly drop-casted from diluted solution on Si/SiO_2 wafers due to the additional advantage in the color change to light blue when flakes are well exfoliated[2]. Herein, GO flakes cast over Si and Si/SiO_2 wafers were visualized with x100 magnification in reflection mode (Nikon Eclipse LV100ND). We used two different wafer types for better visualization and interpretation of the flakes. If the oxidation degree is lower, the deposition on Si wafer allows better visualization of the sample due to less polar surface, thus, less aggregation during drying. Also, the residual GO additives and solvents from the exfoliation and/or washing processes are more visible on the surface of Si/SiO_2 wafers hindering clear imaging (**Figure 1a**).

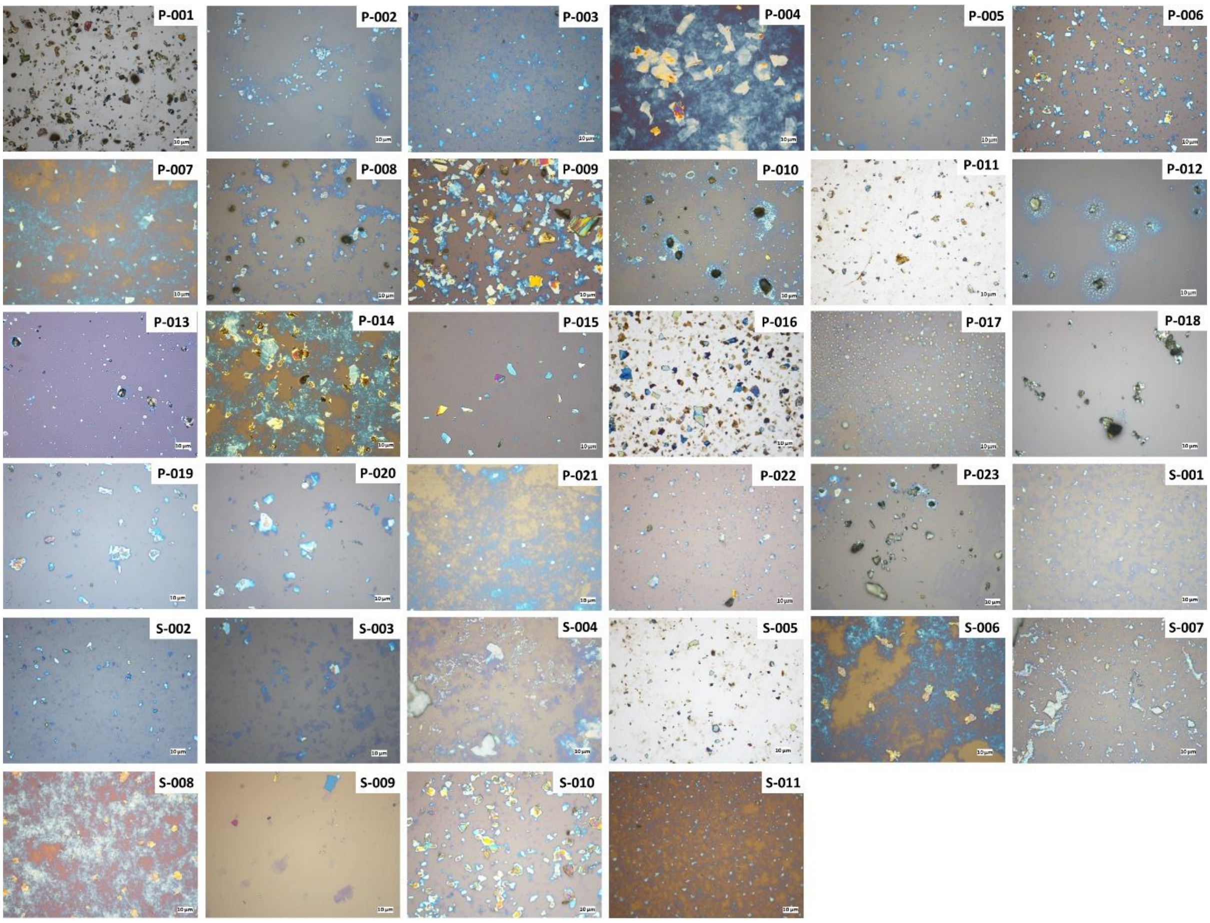

Figure S2. OM images of all samples drop casted from 0.01 mg/mL water suspension after 30 min of ultrasound. Images taken at 100x magnification (scale bar 10 µm).

Most of the samples present at least partially the characteristics of exfoliated GO flakes with different sizes, except for samples P-012, P-013, P-018 and P-023, which were mostly constituted of large aggregates, indicating lack of sufficient oxidation/exfoliation (**Figure S2**).

4. Atomic Force Microscopy (AFM)

Due to its atomic and molecular resolution, Atomic Force Microscopy (AFM) is one of the most well-suited techniques for the study of 2D materials. Here, AFM was used to investigate systematically the thickness of commercial GO samples. Drop-casted GO dispersions on SiO_2/Si substrate with a thickness of 300 nm (except for P-016 and P-019, which were drop-casted on Si substrate) were imaged using a Bruker Dimension FastScan® Atomic Force Microscope (AFM) in a tapping mode with a silicon tip on silicon nitride cantilever (T:

0.6 um, L: 27 µm, W: 32 µm, f_0: 1400 kHz, k: 18 N/m). The images were obtained with a pixel resolution of 512 sample/line. The image processing, height profile, and lateral measurements were performed using the open-source software Gwyddion.

The images were performed in regions of the substrate where well dispersed flakes could be distinguished[3]. All these steps are shown in **Figure S3** using representative OM and AFM images. First, these regions were identified by optical microscopy, followed by AFM with scan size varying from 20 to 2 µm. Finally, the height profiles and the lateral size of each individual flake were measured. For each flake, three different height profile were obtained, and the average thickness was considered. We avoid regions clearly folded, scrolled or overlaid, prioritizing regions that better represent the flake. At least 30 flakes were analyzed per each sample.

A correlation between the thickness and the flake lateral size was also investigated. For this, the lateral size measurements were performed according to the schematic representation below (**Figure S3**). Shortly, the length (L) and width (W) of each individual flake was measured, the area was calculated (W x L), and the flake size was obtained by calculating its square root.

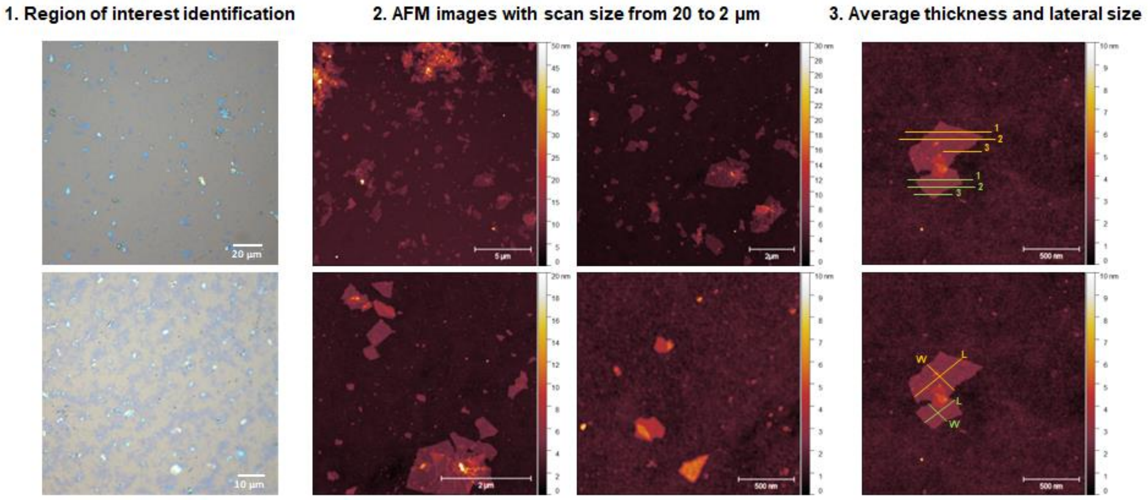

Figure S3. Representative images from samples P-001 and S-001 samples showing the systematically measurement of the thickness and lateral size using AFM.

The AFM measurements and the average thickness are summarized in **Figures S3 and S4,** respectively. Since the GO monolayer thickness can vary depending on the oxidation degree[4] and considering the heterogeneity of the O/C ratio in the samples, the thickness

measurements were not converted in the number of layers. Note, however, that on average the GO monolayer thickness is close to 1 nm and, therefore, we are arbitrarily considering a "thin" GO only the samples with an average thickness of up to 10 nm (**Figure 1e**, highlighted area).

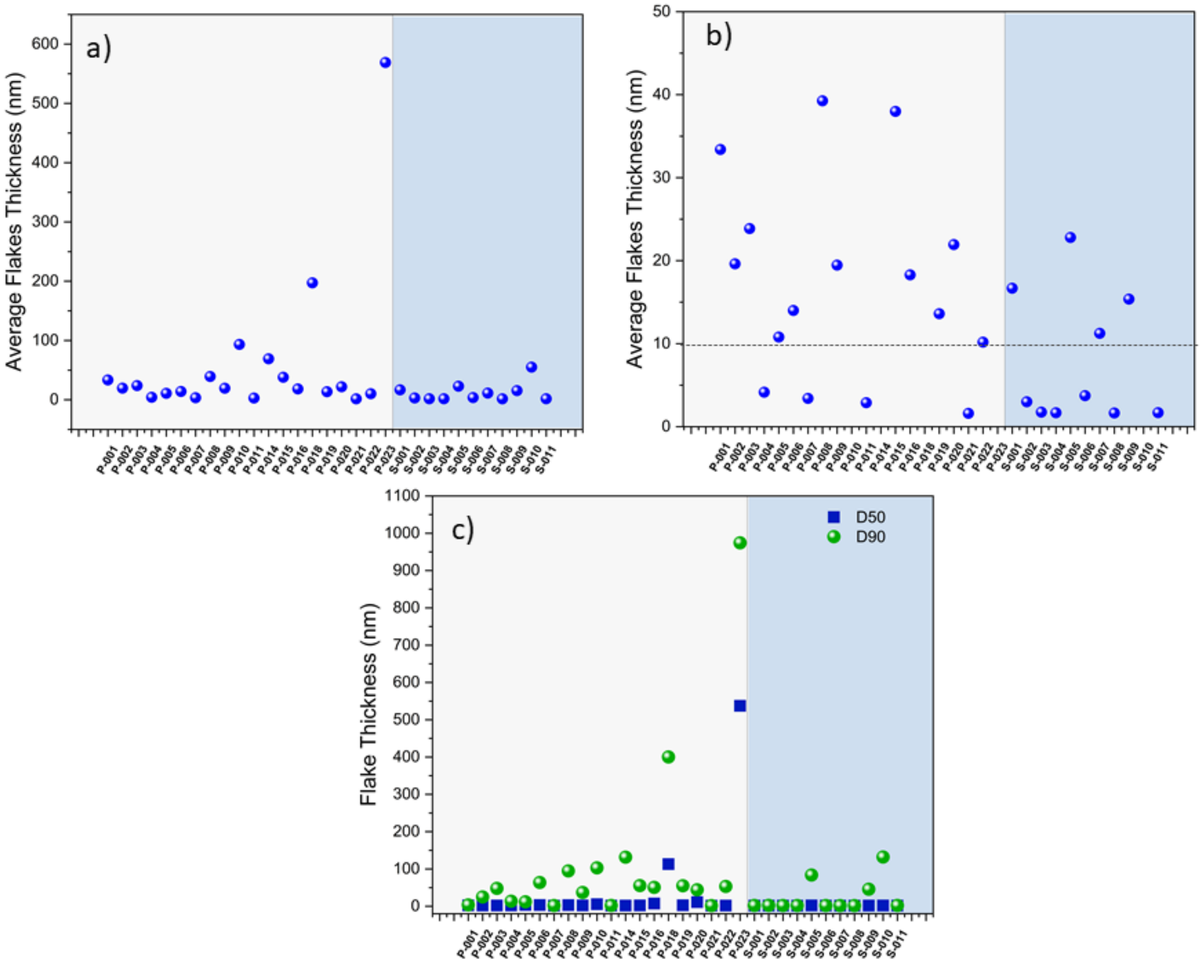

Figure S4. (a) Average flake thickness of all GO samples measured by AFM and (b) zoom in to show only samples with average flake thickness up to 50 nm; (c) GO samples related to thickness (D_{50} and D_{90}) and (d) flake thickness measurements related to the total number of GO samples.

5. Scanning Electron Microscopy (SEM)

0.01 mg/mL GO dispersions were drop-casted on SiO_2/Si substrate and the samples were imaged using FEI Verios 460 L field-emission scanning electron microscope (FESEM) operated at 3 kV. Open-source processing software ImageJ was used for the size

measurement. To identify the GO flakes and measure the surface area, the grayscale SEM raw images were first converted to a binary base (black and white image), where black represents the GO flakes (**Figure S5**). Then, the surface area of at least 200 GO flakes for each sample was measured. To ensure the accuracy of measurement, overlapping flakes and particles that do not appear to be GO flakes were excluded from the analysis. The square root of the area was calculated and the value represents the lateral size of GO flakes. Besides, the surface area measurement cut-off range was set at 0.01 μm^2 to exclude the debris in the samples. As the subject of this paper focuses on the comparison of 2D GO samples, this cut-off range also helped to exclude the carbon fragments with diameters less than 100 nm, which could be the zero-dimensional graphene quantum dots[5].

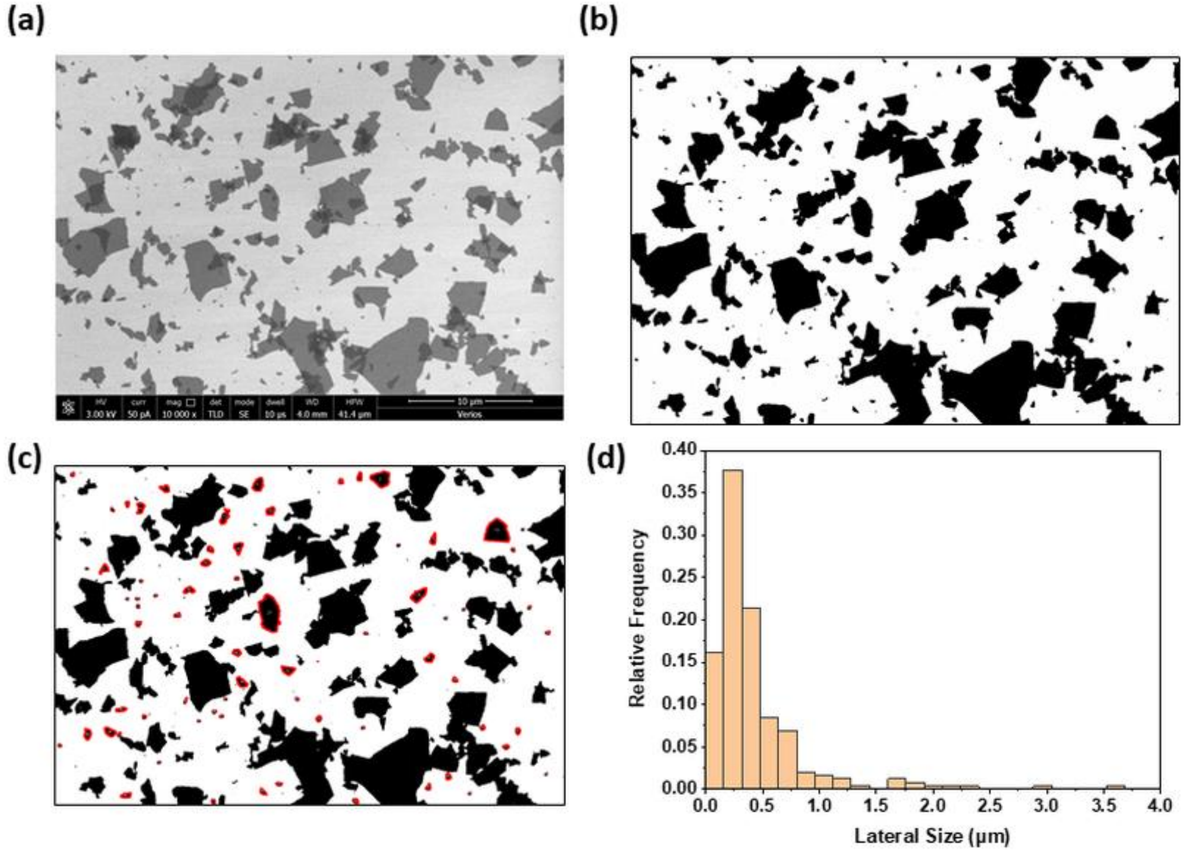

Figure S5. (a) Representative SEM raw image of sample S-003 taken at 10,000× magnification (scale bar: 10 μm) (b) converted to binary and (c) measurement of the surface area of individual GO flakes (highlighted by red outline). (d) The lateral size of a GO flake was obtained by calculating the square root of the surface area and the distribution was presented in the histogram.

According to the ISO guidelines for graphene lateral size measurement, the lateral size of graphene sheets is defined as the mean value of the length and width of the flakes that were measured perpendicularly[6]. This method requires the measurement of at least 200 GO flakes that do not overlap and with typical flake-like morphology.

To date, the characterization method of GO lateral size has not yet been standardized. Over the years, different methods for the measurement of the lateral size of GO flakes have been presented in the literature. For example, the lateral size of GO sheets has been defined as the mean value of the largest and smallest transverse width[7]. In another study, the lateral size of GO sheets was defined as the diameter of an equal-area circle associated with each flake[8].

Based on the collected SEM images, it was observed that most of our samples consisted of a wide range of lateral sizes. It was challenging to measure the length and width of the small-sized flakes individually. In addition, the manual measurement of each flake is highly time-consuming in the case of analyzing a large number of samples. Therefore, software measurement of surface area and calculation of square root of the area of each flake was performed[9]. This method was adopted to ensure the consistent measurement of GO flakes in a wide range of sizes and shapes, without introducing human error. By assuming that GO flakes are squares, a GO flake with an area of 1 μm^2 is corresponding to a lateral size of 1 µm.

Among 34 samples, there was the absence of a typical GO flake-like structure in 6 samples (P-010, P-012, P-013, P-017, P-018, and P-023). Therefore, the size measurement of these samples could not be performed. Based on the observation from OM, GO sheets in P-016 and P-019 were highly aggregated on SiO_2/Si substrates but they were evenly distributed on Si substrate. Therefore, the surface area of GO sheets from P-016 and P-019 was measured using the images taken from the Si substrate. On the other hand, the GO sheets in P-021 and S-011 tend to crumple into clusters, causing fewer open sheets as compared to other samples. Other than plotting the distribution as individual histograms, boxplots of powder and suspension samples have also been plotted for a better comparison among the samples, and the minority larger flakes can be treated as outliers (**Figure S6**)[10].The majority of the samples present an average lateral size < 5 µm. The compilation of all SEM images is presented in **Figure S7**.

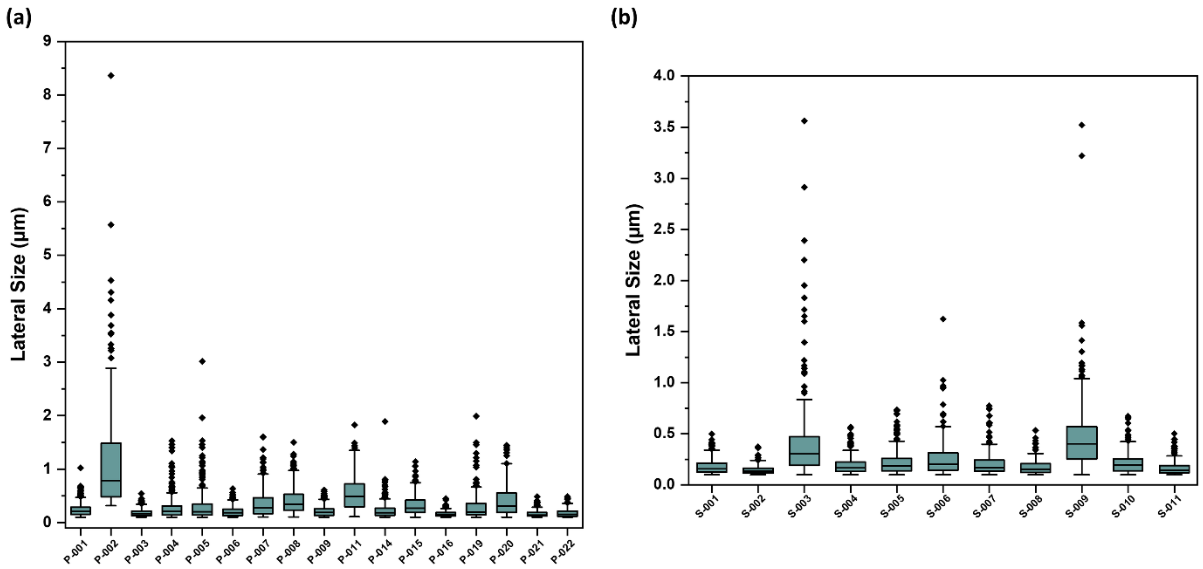

Figure S6. Box plots of the GO flake lateral dimension for (a) powder and (b) suspension samples.

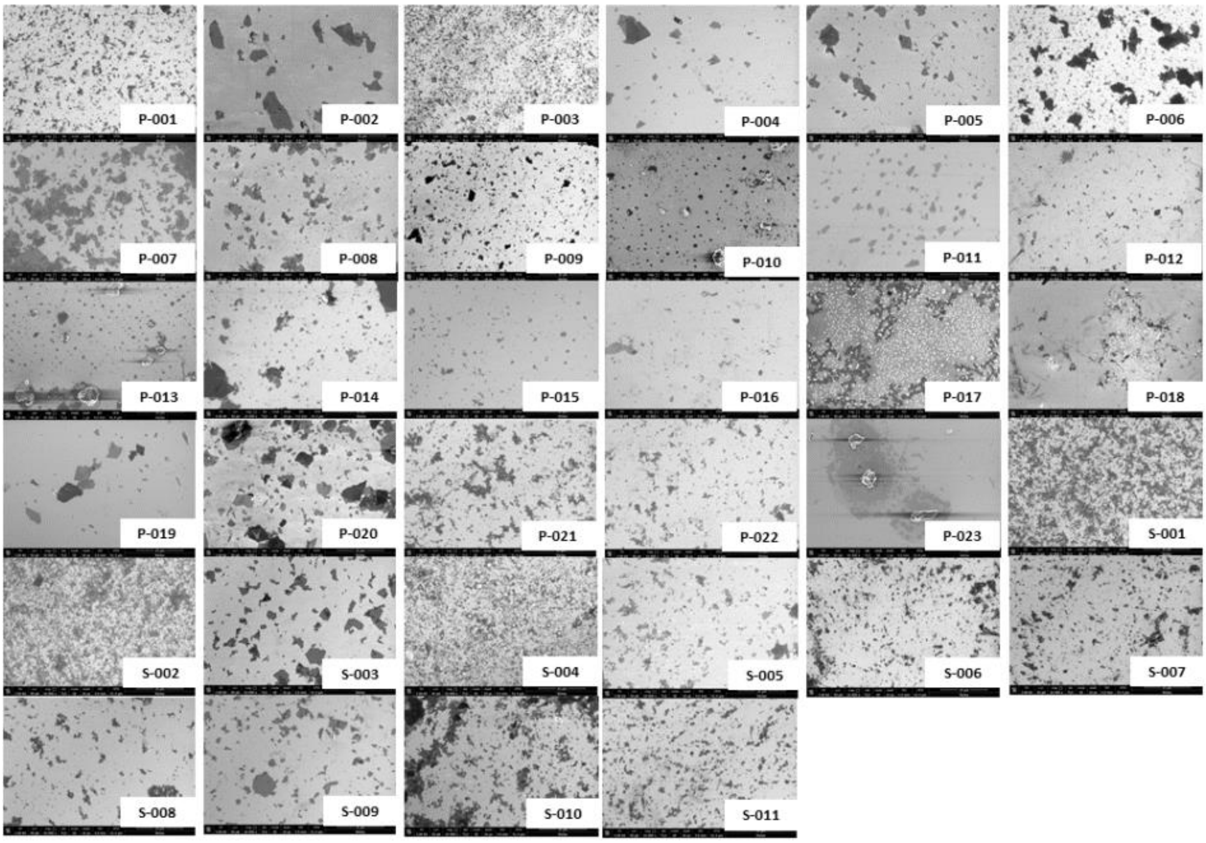

Figure S7. SEM images of all samples casted over Si/SiO_2 (except P-016 and P-019 casted on Si) wafer from 0.01 mg/mL suspensions taken at the same magnification of x10k.

6. X-ray photoelectron spectroscopy (XPS)

X-ray photoelectron spectroscopy (XPS) was performed on a Thermo Escalab 250Xi with an Al anode as the X-ray source (Al-Kα 1486.6 eV). The samples were analyzed as powder. Each spectrum is an average of 5 scans with a pass energy of 150 eV and a step-size of 1 eV for survey spectra, and pass energy of 30 eV, and a step-size of 0.05 eV for high-resolution spectra. An ion gun was used during each scan to neutralize the charging phenomena. To identify and semi-quantify the bands associated with different photo-emitted electrons, we used a previously reported data analysis protocol for fitting[11,12]. The deconvolution of the experimental C1s signal was performed using CasaXPS software, after Shirley's background subtraction[11,12]. Aromatic graphitic carbon (C–C sp^2, located at 284.1 ± 0.3 eV) was fitted as asymmetric Pseudo-Voigt function LF(α, β, *w, m*), where α = 0.65, β =1, *w* = 60 and *m* = 150, with FWHM = 0.82 ± 0.02 eV and Γ = 0.14. Symmetric pseudo-Voigt function (50% Gaussian and 50% Lorentzian) was used to fit the remaining bands, which correspond to the aliphatic carbon (mainly C–C sp^3, 285.0 ± 0.2 eV), carbons bound to hydroxyl (C–OH, 285.7 ± 0.2 eV), epoxide groups (C–O–C, 286.7 ± 0.2 eV), carbonyl groups (C=O, 288.0 ± 0.3 eV), carboxyl groups (-C–OOH, 289.0 ± 0.3 eV), and the contribution of the plasmonic transition (secondary peak π–π, 291.5 ± 0.4 eV). The C1s overall signal is assumed as the sum of the C1s separate sub-peaks related to each C-based functionality present in the sample, and the total area of all the C1s peaks is proportional to the total number of C species present in the sample. The area of each sub-peak (Ai) is, therefore, directly proportional to the number of C-atoms in each functional group, and the oxygen-to-carbon ratios for each functionality are: (1:1) for hydroxyl, (1:2) for epoxy, (1:1) for carbonyl, and (2:1) for carboxyl. Thus, the O/C ratio is given by the equation:

$$\frac{O}{C}_{fit} = \frac{A_{C-OH} + \frac{1}{2} \cdot A_{C-O-C} + A_{C=O} + 2 \cdot A_{O-C=O}}{A_{total}}$$

This data-analysis protocol, which has been developed and improved with extensive studies on the XPS signals from C-based materials, is preferred against the conventional-area-method since it accounts for critical artefacts, such as the influence of the substrate and adsorbed water. All C1s spectra are presented in **Figure S8**, and the calculated percentages of each functional group and O/C ratio are gathered in **Table S1.**

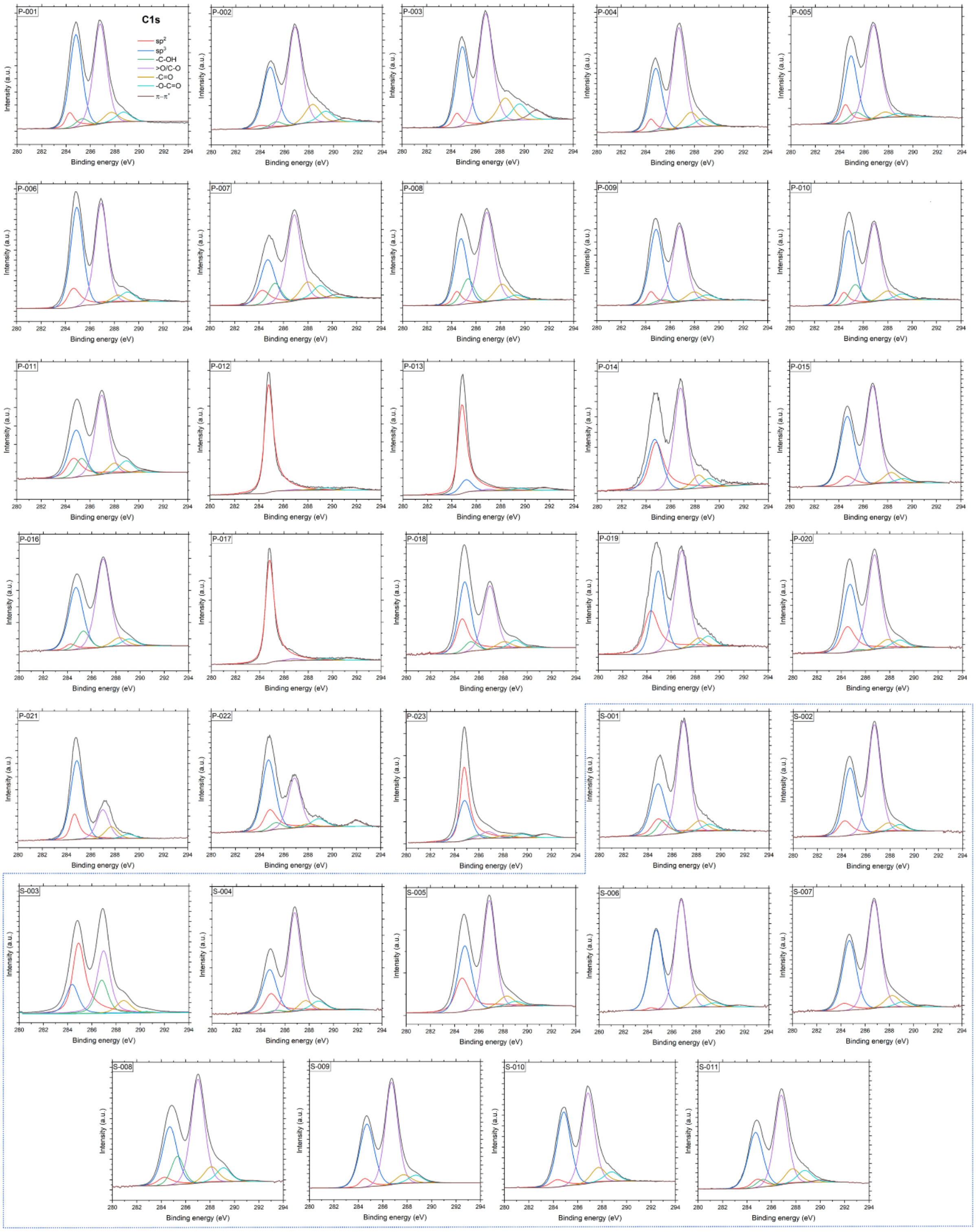

Figure S8. C1s spectra of all samples.

Table S1. Calculated percentages of functional groups and O/C ratios obtained from XPS data.

Sample	sp^2	sp^3	C-OH	>O/C-O	C=O	OC=O	O/C[1]	O/C[2]	π-π*
**P-001**	5.90	38.28	3.05	43.38	4.80	4.60	0.38	0.51	0.00
**P-002**	2.18	30.80	2.31	48.35	9.23	5.44	0.47	0.62	1.69
**P-003**	4.69	29.28	0.00	45.52	9.68	6.88	0.42	0.56	3.94
**P-004**	5.57	30.68	0.92	49.86	8.21	4.48	0.48	0.64	0.29
**P-005**	8.01	30.87	5.05	50.72	2.77	1.65	0.36	0.48	0.93
**P-006**	10.23	40.72	0.00	41.04	2.76	4.55	0.42	0.56	0.70
**P-007**	9.50	22.87	8.81	41.77	8.74	6.45	0.46	0.61	1.85
**P-008**	5.41	28.32	10.68	44.82	8.04	2.38	0.41	0.54	0.34
**P-009**	6.21	40.64	2.24	42.07	5.20	3.21	0.39	0.52	0.43
**P-010**	5.64	35.74	9.14	40.18	5.19	3.34	0.35	0.46	0.77
**P-011**	13.56	26.14	8.56	39.89	4.71	5.97	0.38	0.50	1.16
**P-012**	92.77	0.00	0.00	0.78	0.94	2.37	0.05	0.07	3.15
**P-013**	79.86	12.25	0.00	1.35	0.83	2.34	0.05	0.07	3.36
**P-014**	27.2	22.7	0.00	39.4	5.27	3.94	0.36	0.49	1.45
**P-015**	6.57	36.96	0.00	48.04	5.60	2.24	0.44	0.58	0.59
**P-016**	3.12	33.62	8.21	46.75	4.49	3.59	0.41	0.55	0.22
**P-017**	92.75	0.00	0.00	1.91	0.38	2.03	0.19	0.26	2.93
**P-018**	20.48	34.08	4.57	32.79	3.68	3.70	0.38	0.51	0.70
**P-019**	23.09	29.92	0.00	39.91	3.11	3.94	0.38	0.51	0.00
**P-020**	15.40	31.03	0.57	45.02	4.01	3.64	0.43	0.57	0.33
**P-021**	15.56	53.87	0.33	19.34	7.81	2.87	0.21	0.28	0.22
**P-022**	15.28	41.95	3.72	28.67	1.94	5.16	0.41	0.55	3.27
**P-023**	51.55	30.65	2.90	5.85	2.60	3.24	0.06	0.08	3.21
**S-001**	10.46	25.68	6.24	48.82	5.25	3.34	0.43	0.57	0.21
**S-002**	9.68	33.06	0.00	49.63	4.38	3.26	0.44	0.59	0.00
**S-003**	11.76	34.99	2.11	40.55	4.26	5.88	0.46	0.61	0.45
**S-004**	13.50	25.06	1.14	49.66	4.99	5.22	0.45	0.60	0.42
**S-005**	20.15	29.16	0.00	43.43	4.72	2.20	0.39	0.53	0.34
**S-006**	1.00	39.43	0.00	49.72	6.81	2.15	0.44	0.59	0.90
**S-007**	4.79	34.56	0.00	51.30	5.92	2.66	0.43	0.58	0.76
**S-008**	4.78	26.79	11.12	43.40	7.03	6.39	0.45	0.60	0.51
**S-009**	4.60	35.71	0.00	50.16	4.90	4.33	0.46	0.61	0.30
**S-010**	5.00	37.91	0.07	43.78	7.91	5.11	0.45	0.60	0.22
**S-011**	5.20	30.87	3.54	44.45	8.02	6.76	0.50	0.66	1.17

[1] atomic ratio. [2] mass ratio.

7. Elemental analysis (EA)

Elemental analysis to quantify carbon, nitrogen, hydrogen, sulphur, and oxygen (CNHS-O) was performed using an organic elemental analyzer Vario El cube (Elementar – Germany). All the data is gathered in **Table S2**.

Table S2. Elemental analysis C, H, N, S, O as well as the O/C ratios for all the samples.

Sample	C %	H %	N %	S %	O %	O/C[1]	O/C[2]	Total %
**P-001**	45.43	2.62	0.87	1.86	49.38	0.82	1.09	100.2
**P-002**	43.07	2.85	1.09	1.05	51.45	0.90	1.19	99.5
**P-003**	44.87	2.48	0.88	2.33	49.41	0.83	1.10	100.0
**P-004**	43.98	2.67	0.92	1.98	51.54	0.88	1.17	101.1
**P-005**	52.58	2.27	1.34	1.35	43.20	0.62	0.82	100.7
**P-006**	46.60	2.60	1.62	1.99	48.67	0.78	1.04	101.5
**P-007**	44.68	2.86	1.15	0.85	47.68	0.80	1.07	97.2
**P-008**	45.78	2.40	1.72	1.81	49.07	0.80	1.07	100.8
**P-009**	46.36	2.59	2.38	1.57	47.12	0.76	1.02	100.0
**P-010**	64.41	1.54	2.05	0.56	31.85	0.37	0.49	100.4
**P-011**	49.54	2.55	0.06	0.47	42.17	0.64	0.85	94.8
**P-012**	96.68	0.24	0.11	0.25	3.20	0.03	0.03	100.5
**P-013**	94.40	0.30	0.14	0.30	4.45	0.04	0.05	99.6
**P-014**	48.44	2.54	0.00	0.44	42.59	0.66	0.88	94.0
**P-015**	44.93	2.49	0.07	1.96	41.36	0.69	0.92	90.8
**P-016**	45.98	2.59	0.06	2.27	42.79	0.70	0.93	93.7
**P-017**	78.30	0.54	0.09	3.44	10.88	0.10	0.14	93.3
**P-018**	50.67	2.33	0.07	3.09	38.42	0.57	0.76	94.6
**P-019**	45.41	2.75	0.05	1.48	44.21	0.73	0.97	93.9
**P-020**	43.15	2.74	0.05	2.83	44.41	0.77	1.03	93.2
**P-021**	42.94	2.90	0.05	1.73	46.48	0.81	1.08	94.1
**P-022**	39.90	2.75	0.10	5.10	43.48	0.82	1.09	91.3
**P-023**	85.77	0.89	0.09	0.46	9.04	0.08	0.11	96.3
**S-001**	46.05	3.01	0.00	0.47	38.22	0.62	0.83	87.8
**S-002**	46.12	2.75	0.00	1.64	42.66	0.69	0.92	93.2
**S-003**	42.07	3.04	2.60	3.17	42.94	0.77	1.02	93.8
**S-004**	43.63	2.88	0.09	2.08	45.85	0.79	1.05	94.5
**S-005**	48.29	2.75	0.09	1.12	39.81	0.62	0.82	92.1
**S-006**	43.14	3.02	0.11	2.22	44.45	0.77	1.03	92.9
**S-007**	44.33	2.90	0.67	2.05	42.87	0.73	0.97	92.8
**S-008**	40.65	3.11	0.00	2.23	48.87	0.90	1.20	94.9
**S-009**	43.85	2.90	0.00	2.42	45.67	0.78	1.04	94.8
**S-010**	46.02	2.60	0.97	1.40	41.99	0.68	0.91	93.0
**S-011**	41.21	2.92	1.48	2.92	45.74	0.83	1.11	94.3

[1] atomic ratio. [2] mass ratio.

8. Raman Spectroscopy

Raman spectroscopy has been extensively used as a non-destructive characterization technique to evaluate the structural organization of graphene and its derivatives. Briefly, it allows an understanding of how a monochromatic laser impacts the molecular vibrational modes and phonons due to inelastic scattering shifts. The Raman spectra collected provide detailed information about disorders, edges, thickness, doping, as well as the thermal conductivity of such carbon-related materials under different conditions[13].

Among the main Raman features found in a typical carbon-based material are the D (1350 cm^{-1}), G (1590 cm^{-1}), and 2D (2680 cm^{-1}) bands. The first-order G and D bands arise from the vibration of sp^2-hybridized carbon materials. The former corresponds to E_2g phonons from stretching of sp^2 carbon pairs located at the center of Brillouin zone, whereas the latter is attributed to the breathing mode of aromatic rings and its intensity is a defects indicator. The overtone of the D band, also known as the 2D band, is correlated to the double resonance transitions of phonons with opposite momentum. Different from the D band, which is only visible when the sample has defects, the 2D band is active regardless of any disorders due to the inelastic scattering of second-order phonons[14].

0.01 mg/mL GO suspensions were deposited on SiO_2/Si substrate fixed on a microscope slide and analyzed using Raman WITEC Alpha 300R with 100x magnification and 532 nm laser wavelength. At least three different regions were imaged via OM and subsequently evaluated in the oscilloscope mode to obtain at least 50 spectra from each sample (0.5 s integration time and 10 accumulations). From those, 20 spectra per sample were chosen for minimal background noise and were normalized for the G band intensity. The correspondent D, G, and 2D bands were identified accordingly. Additionally, the I_D/I_G and I_G/I_{2D} ratios were calculated (**Table S3**).

Table S3. D, G, and 2D peaks positions along with the I_D/I_G and I_G/I_{2D} for all the samples.

Sample	D	G	2D	I_D/I_G	I_G/I_{2D}
**P-001**	1350.6 ± 2.1	1601.5 ± 1.8	2711.0 ± 12.1	1.05 ± 0.01	9.38 ± 3.67
**P-002**	1357.8 ± 3.9	1603.0 ± 2.3	2711.9 ± 26.2	0.98 ± 0.08	5.96 ± 3.21
**P-003**	1348.7 ± 2.6	1601.0 ± 5.3	2699.7 ± 8.1	1.11 ± 0.13	18.93 ± 5.61
**P-004**	1345.5 ± 3.6	1598.3 ± 3.8	2692.2 ± 8.1	1.05 ± 0.01	20.93 ± 6.26
**P-005**	1343.5 ± 4.8	1598.1 ± 6.8	2675.1 ± 12.8	1.19 ± 0.11	21.39 ±18.97
**P-006**	1344.2 ± 5.4	1599.7 ± 6.6	2677.6 ± 11.1	1.19 ± 0.11	17.37 ± 9.54
**P-007**	1355.5 ± 3.4	1602.5 ± 2.5	2719.0± 8.9	1.01 ± 0.03	6.83 ± 4.46
**P-008**	1348.7 ± 1.4	1603.9 ± 2.1	2699.4 ± 8.2	1.06 ± 0.01	16.16 ± 8.42
**P-009**	1350.3 ± 1.5	1601.4 ± 1.7	2699.2 ± 6.7	1.06 ± 0.01	15.31 ± 8.67
**P-010**	1348.4 ± 2.6	1587.2 ± 6.8	2686.4 ± 12.1	1.05 ± 0.12	29.46 ± 23.57
**P-011**	1351.6 ± 3.3	1599.2 ± 3.3	2699.9 ± 19.1	1.04 ± 0.09	5.49 ± 3.51
**P-012**	1354.5 ± 3.5	1585.8 ± 4.3	2708.6 ± 7.8	0.33 ± 0.17	2.69 ± 0.74
**P-013**	1352.5 ± 4.0	1608.5 ± 121.5	2701.0 ± 8.5	0.45 ± 0.21	3.65 ± 0.53
**P-014**	1345.1 ± 2.3	1594.27 ± 6.9	2687.1 ± 7.6	1.06 ± 0.06	41.47 ± 17.78
**P-015**	1344.9 ± 2.3	1592.85 ± 6.9	2684.0 ± 6.8	1.01 ± 0.11	31.62 ± 48.69
**P-016**	1345.9 ± 1.8	1593.4 ± 2.9	2695.2 ± 5.2	1.04 ± 0.02	24.94 ± 12.74
**P-017**	1352.8 ± 4.8	1597.9 ± 0.0	-	0.91 ± 0.05	-
**P-018**	1349.2 ± 2.6	1585.6 ± 9.1	2697.7 ± 8.8	0.78 ± 0.39	8.02 ± 4.28
**P-019**	1350.1 ± 2.4	1596.5 ± 2.9	2694.3 ± 6.8	1.07 ± 0.03	25.79 ± 17.38
**P-020**	1349.5 ± 1.9	1597.8 ± 4.4	2697.2 ± 4.8	1.07 ± 0.03	14.09 ± 10.68
**P-021**	1346.9 ± 2.8	1601.1 ± 3.7	2691.9 ± 6.7	1.02 ± 0.06	13.36 ± 6.81
**P-022**	1348.1 ± 2.6	1605.3 ± 2.0	2694.8 ± 16.2	1.09 ± 0.05	14.38 ± 13.86
**P-023**	1350.5 ± 4.3	1589.00 ± 5.5	2693.7 ± 17.6	1.06 ± 0.15	72.47 ± 45.49
**S-001**	1349.4 ± 3.3	1601.4 ± 2.8	2705.2 ± 10.8	1.04 ± 0.02	6.60 ± 1.27
**S-002**	1351.9 ± 3.1	1602.4 ± 5.7	2693.2 ± 5.8	1.09 ± 0.08	10.32 ± 1.94
**S-003**	1351.0 ±2.9	1594.4 ± 8.9	2695.3 ± 8.9	1.11 ± 0.13	9.69 ± 3.54
**S-004**	1351.2 ± 2.0	1601.9 ± 3.6	2715.2 ± 12.4	1.01 ± 0.04	5.22 ± 1.02
**S-005**	1346.7 ± 2.3	1598.0 ± 1.8	2686.4 ± 4.4	1.13 ± 0.01	8.88 ± 0.98
**S-006**	1346.9 ± 1.5	1590.4 ± 3.8	2694.5 ± 7.3	1.07 ± 0.02	22.93 ± 9.61
**S-007**	1349.7 ± 2.0	1603.4 ± 2.0	2701.7 ± 7.8	1.04 ± 0.07	6.16 ± 0.62
**S-008**	1352.3 ± 0.0	1600.1 ± 4.6	2706.0 ± 6.1	1.01 ± 0.02	18.91 ± 5.71
**S-009**	1348.3 ± 1.7	1590.7 ± 4.1	2693.2 ± 6.7	1.07 ± 0.03	28.83 ± 13.75
**S-010**	1349.7 ± 2.4	1596.4 ± 6.2	2693.2 ± 5.7	1.09 ± 0.05	25.75 ± 16.71
**S-011**	1350.1 ± 2.7	1604.5 ± 2.7	2706.9 ± 5.8	1.03 ± 0.01	8.64 ± 1.76

Several dark spots without GO Raman signal were detected over random regions for distinct samples. Nonetheless, it is important to highlight that this is not a trend, since some of these dark spots show the characteristic GO bands.

The Raman spectra of 20 out of 34 GO samples show D and G bands with strong intensity, which are characteristic of GO samples with small crystal sizes[14]. None of the

samples presented D’ peak. **Figure S9** shows some representative OM images and the Raman spectra collected for two samples (S-005 in **Figure 9**, and S-009 in **Figure 9b**). The prominent Raman signals are seen at 1345-1347 cm^{-1} (D) and 1590-1605 cm^{-1} (G), respectively. The 2D bands shown as broad and weak bands at 2682-2704 cm^{-1} have been previously attributed as an additional indicator of disorders in GO samples, and another defect-activated band is visible near 2900 cm^{-1} [14].

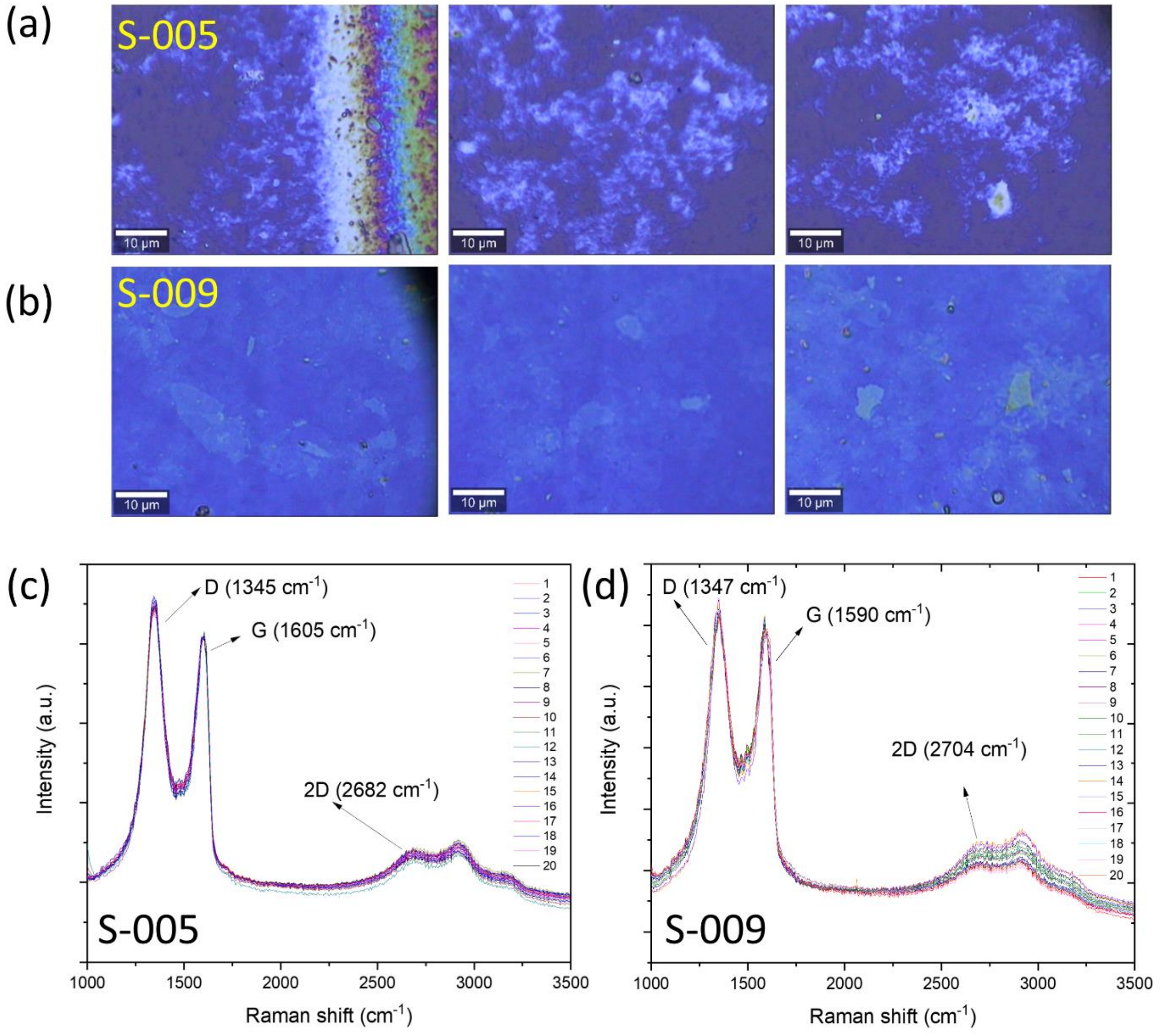

Figure S9. (a, b) Representative OM images, and (c, d) Raman spectra for GO samples with characteristic GO Raman profile.

The ratio of peak intensities of D and G Raman bands (I_D/I_G) is used to estimate the density of structural disorder in graphene-based samples. The disorder level can be understood in terms of low or high defect densities, for which the I_D/I_G ratio can be increased

or decreased, respectively[14]. The I_D/I_G ratios calculated for samples P-012, P-013, and P-018 are considerably lower compared to the other GO samples, of the order of 0.33 ± 0.17, 0.45 ± 0.21, and 0.78 ± 0.39, respectively. These results corroborate the SEM data interpretation (see Section 5). Additionally, in **Figure S10** it can be seen the absence of a typical GO flake-like structure (**Figures S10 a and b**) and also atypical Raman profile spectra (**Figures S10c and d**) for samples P-012 and P-013.

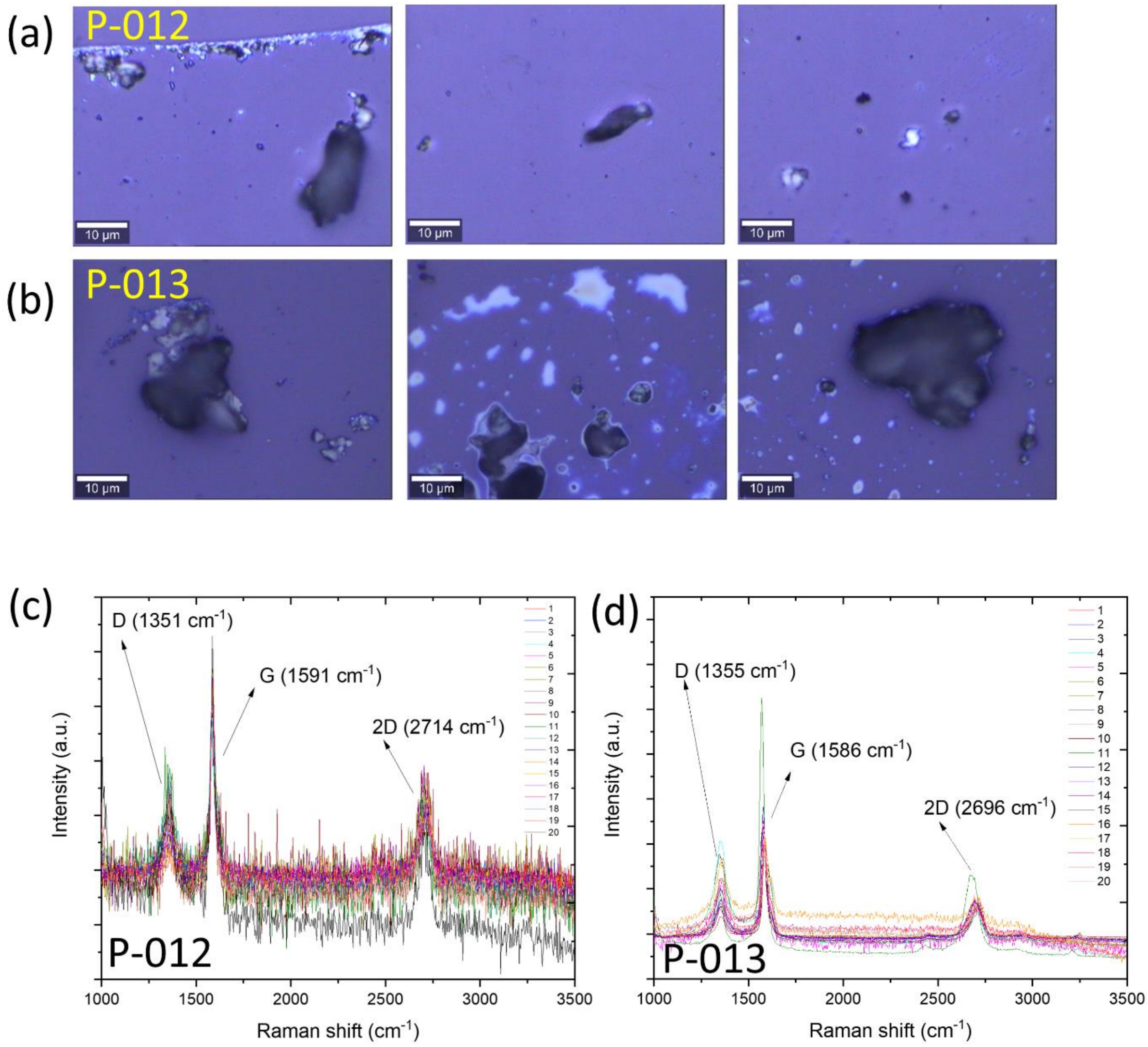

Figure S10. (a, b) Representative OM images, and (c, d) Raman spectra for GO samples with non-characteristic GO Raman profile.

In particular, the typical GO bands were not identified at sample P-017 at the expected Raman shift range. As such, different substrates (Si and Si/SiO_2) were analyzed, but GO flakes

still could not be localized (see **Figure S11a**). Overall, only two Raman spectra were successfully acquired for this sample, as can be seen in **Figure S11b**.

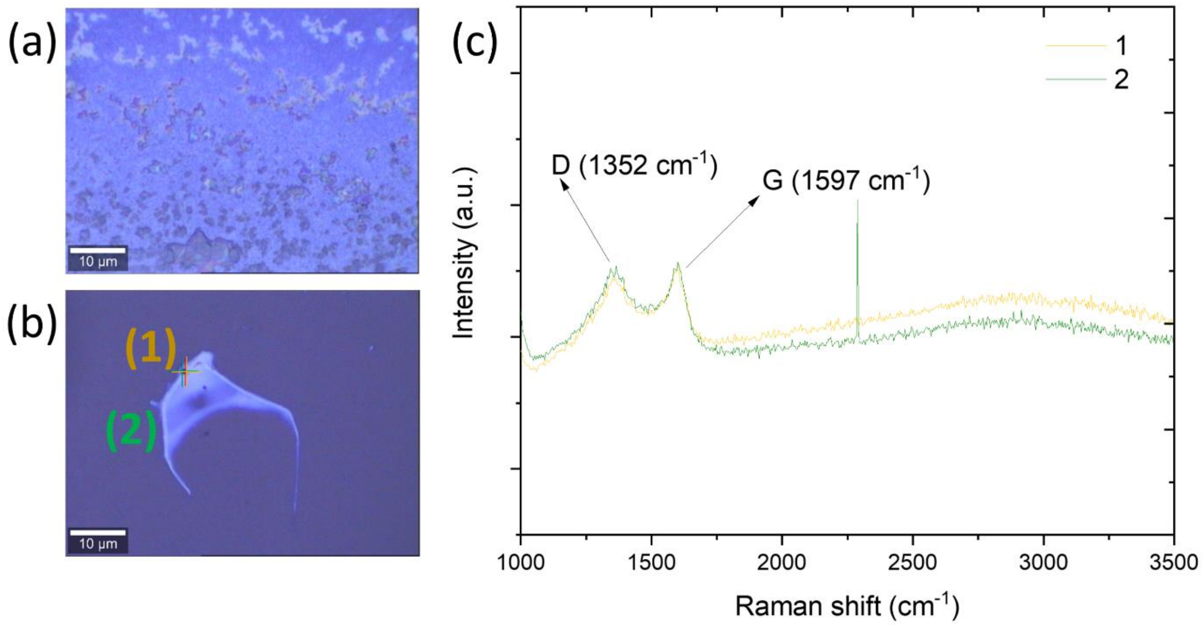

Figure S11. (a, b) OM images, and (c) Raman spectra for GO sample P-017.

9. Thermogravimetric analysis (TGA)

Thermogravimetry is a powerful and reliable tool for the qualitative and quantitative analysis of graphene materials. The few-layer graphene (FLG), GO, reduced graphene (rGO), and graphite show very distinctive thermal decomposition behavior. The characteristic parameters such as thermal stability (the temperature at which the material starts to decompose), maximum thermal degradation peak position, and shape are all related to their intrinsic chemical and physical structures and can be easily identified[15].

To perform the analyses, powder or freeze-dried samples were placed in a desiccator for a minimum of 24 h before the measurement. The thermogravimetric analyses (TGA) were performed on a Netzsch STA 449 Jupiter. An average sample weight of 6 mg was placed in a ceramic sample holder and heated at 10 °C/min from 50 to 1000 °C under a constant synthetic airflow (50 mL/min). The first derivative of the weight to temperature (DTG) was obtained and by using Gaussian multiple peak fit maximum degradation temperature rates (T_d) were calculated for each sample (**Table S4**). **Figures S12 a, b, and c** demonstrate all TGA curves.

Table S4. Thermal degradation temperature regions with maximum thermal degradation rates obtained from the DTG curves.

Sample	T_{d1} (˚C)	T_{d2} (˚C)	T_{d3} (˚C)	Sample	T_{d1} (˚C)	T_{d2} (˚C)	T_{d3} (˚C)
**P-001**	-	200	470*	**P-018**	53	196	555*
**P-002**	-	-	-	**P-019**	73	209	524*
**P-003**	-	198	493*	**P-020**	62	198	527*
**P-004**	-	199	509*	**P-021**	71	197	545*
**P-005**	59	243*	539	**P-022**	79*	166, 277	532
**P-006**	-	178	544*	**P-023**	-	-	569*
**P-007**	-	212*	494	**S-001**	-	-	-
**P-008**	-	202	483*	**S-002**	63	188	510*
**P-009**	89	203*	545	**S-003**	51	182	488*
**P-010**	-	230	572*	**S-004**	-	-	-
**P-011**	51	222*	607	**S-005**	72	206	412*
**P-012**	-	-	730*	**S-006**	80	204	475*
**P-013**	-	-	658*	**S-007**	53	199	499*
**P-014**	74	220*	608	**S-008**	75	182	585*
**P-015**	-	207*	614	**S-009**	-	191	476*
**P-016**	-	202*	614	**S-010**	68	206	476*
**P-017**	-	-	813*	**S-011**	62	202	547*

*maximum degradation rate temperature.

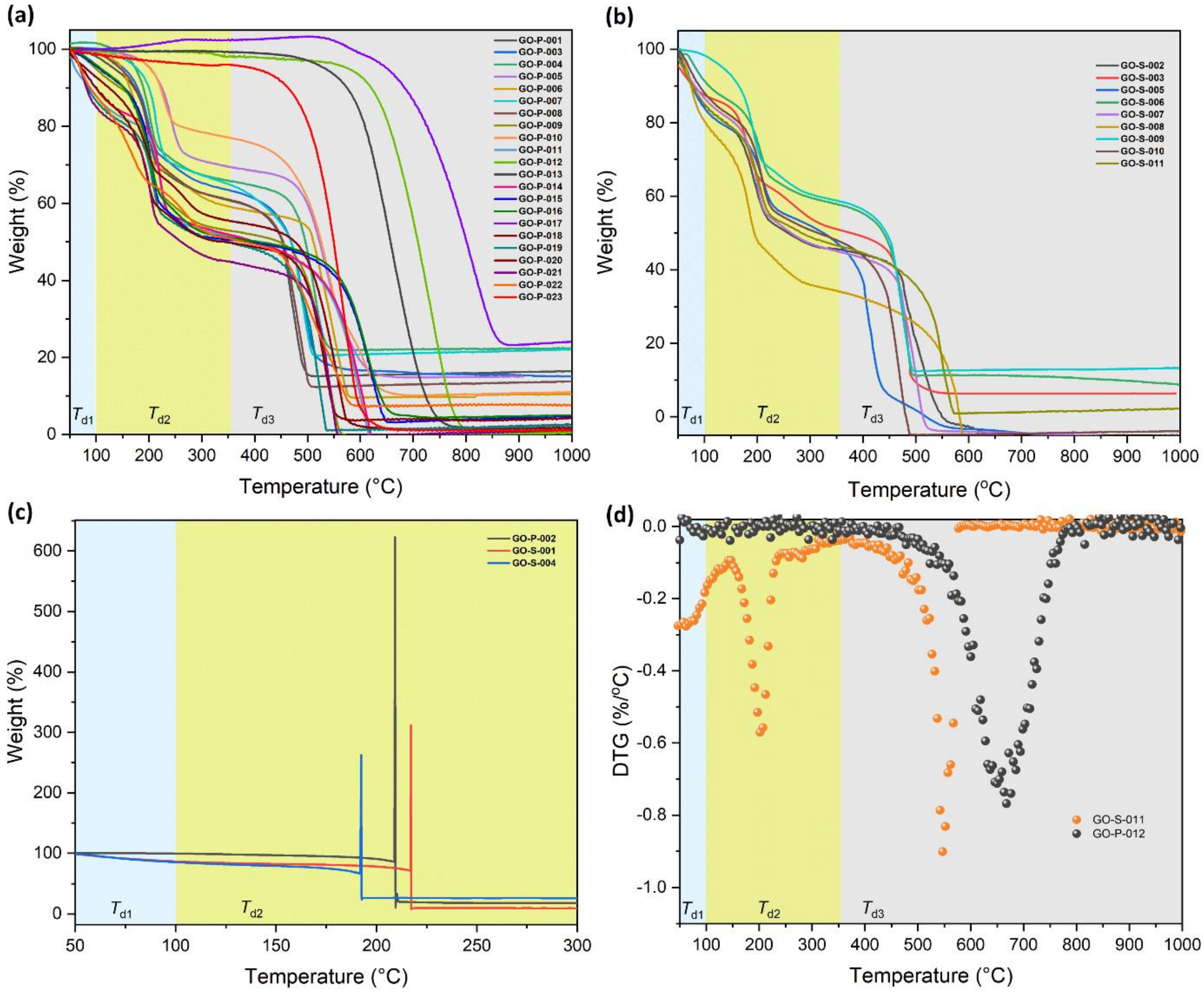

Figure S12. (a, b, and c) thermogravimetric curves of all samples were obtained at 10 °C/min from 50 to 1000 °C under a constant synthetic airflow (50 mL/min). (d) Representative comparison of DTG curves for typical GO (S-011) and non-oxidized sample (P-012).

The typical GO TGA curve presents three characteristic mass loss steps. First (T_{d1}, below 100 °C) is related to water evaporation. The second (T_{d2},100–360 °C) is caused by the oxygen-containing groups' decomposition, and the third step (T_{d3}, 360–1000 °C) is induced by carbon combustion[15]. Most of the analyzed samples demonstrate a typical three degradation-step behavior varying the level and type of oxygenated groups. Samples P-002, S-001, and S-004 showed abrupt degradation at ~200 °C (**Figure S12c**). On the other hand samples P-012, P-013, P-017, and S-003 show one or two degradation steps, in the regions more characteristic for rGO and graphene rather than GO. **Figure S12d** demonstrates a comparison between highly oxidized S-011 and basically non-oxidized P-012, confirming that TGA is a quick and easy process of verifying the GO quality.

10. X-ray diffraction measurements (XRD)

X-ray diffraction measurements (XRD) were performed on a powder diffractometer (Rigaku Miniflex 600) equipped with a Bragg-Brentano $\theta : 2\theta$ goniometer, using Cu K-α radiation (1.5406 Å. The scan parameters were kept the same for all the samples; 2θ range from 3° to 50°, stepwise at 0.05 °/step, and integration time of 2 s/step. For minimizing the effects water and other solvents that could be residual from the synthesis process, the samples were all dried and kept in a desiccator for more than 24h. Also, to ensure the quality of the signal and minimize the loss of scattering power due to sample roughness, the powder was pressed into a continuous film and then loaded onto a low-background sample holder. The interlayer distance values were calculated from the centroid position of each peak fitted using pseudo-Voigt as a profile function. **Figure S13** demonstrates XRD plots for all measured samples.

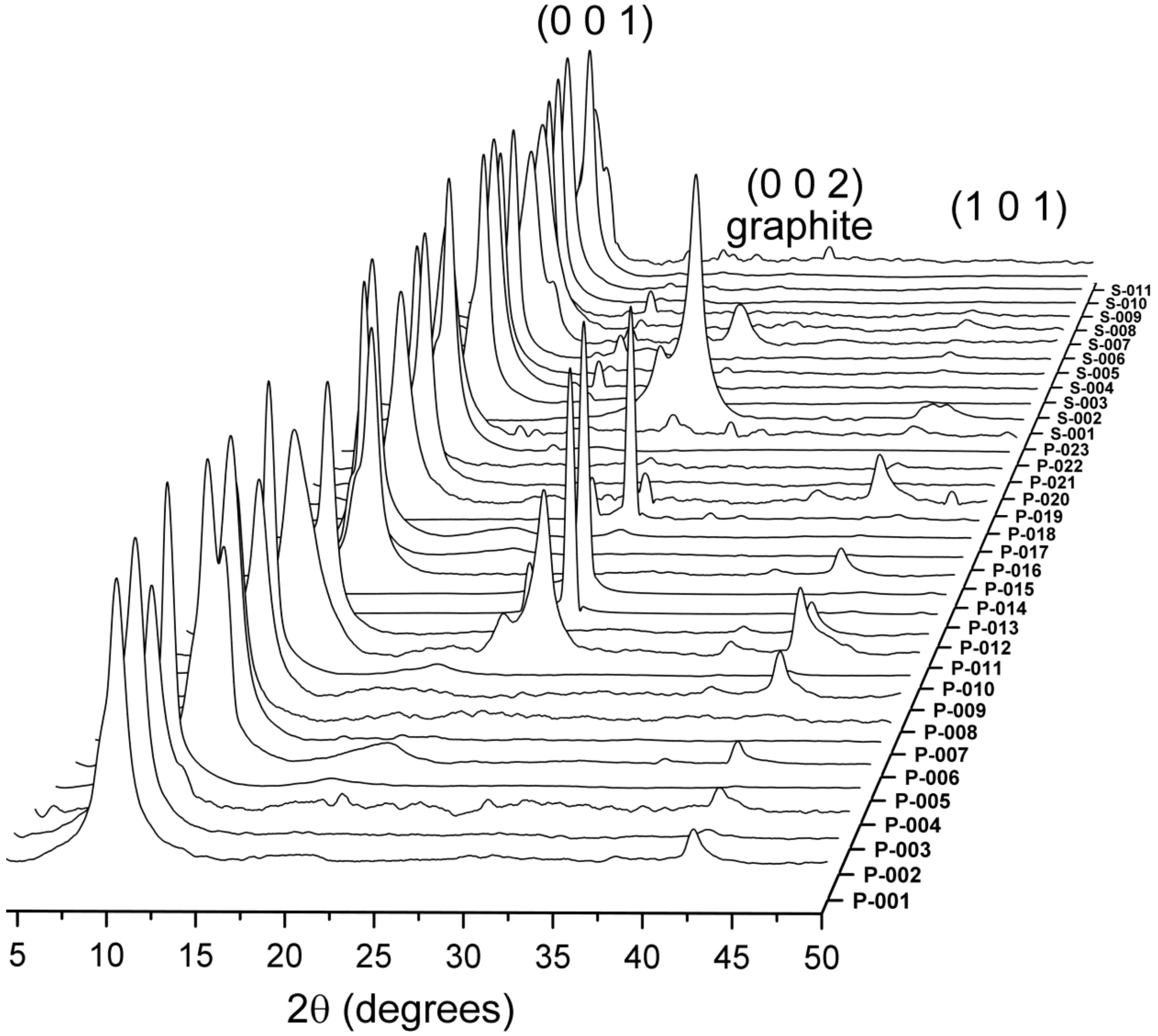

Figure S13. Normalized XRD patterns of all samples.

11. Infrared Spectroscopy

Since GO contains hydroxyl, epoxide, and carboxyl functional groups, it can be qualitatively identified with the infrared spectroscopy. All dry GO samples were firstly analyzed using attenuated total reflectance (ATR) infrared spectroscopy to identify chemical bonds (**Figure S14, Table S6**). Samples with unusual spectra (noise/untraceable peaks) were additionally screened using Fourier-transform infrared (FTIR) transmission microscopy to conclude the result (**Table S7**).

A typical GO spectrum presents peaks at 3412-3420 cm^{-1} (O-H stretching), 1720-1740 cm^{-1} (C=O stretching), 1590-1627 cm^{-1} (carbon backbone C=C stretching), 1356-1365 cm^{-1} (C-OH stretching), 1225-1260 cm^{-1} (C-O-C stretching), 1056-1078 cm^{-1} (C-O stretching)[16,17]. The peaks are represented as ranges rather than the single values, since the peak position shifts with synthesis method and content of contaminants. The broad peak at ca. 3420 cm^{-1} indicates presence of -OH groups that could be attributed to the hydroxyl groups of GO as well as to water molecules adsorbed to hydrophilic GO. Presence of water also impacts the intensity of the peak at 1590-1620 cm^{-1} owing to its bending vibrations. Additionally, GO spectrum can present sulfonic acid groups -SO_3H (1175 cm^{-1} and 1126 cm^{-1} for S-O, and 1040 cm^{-1} for S-phenyl).[16]

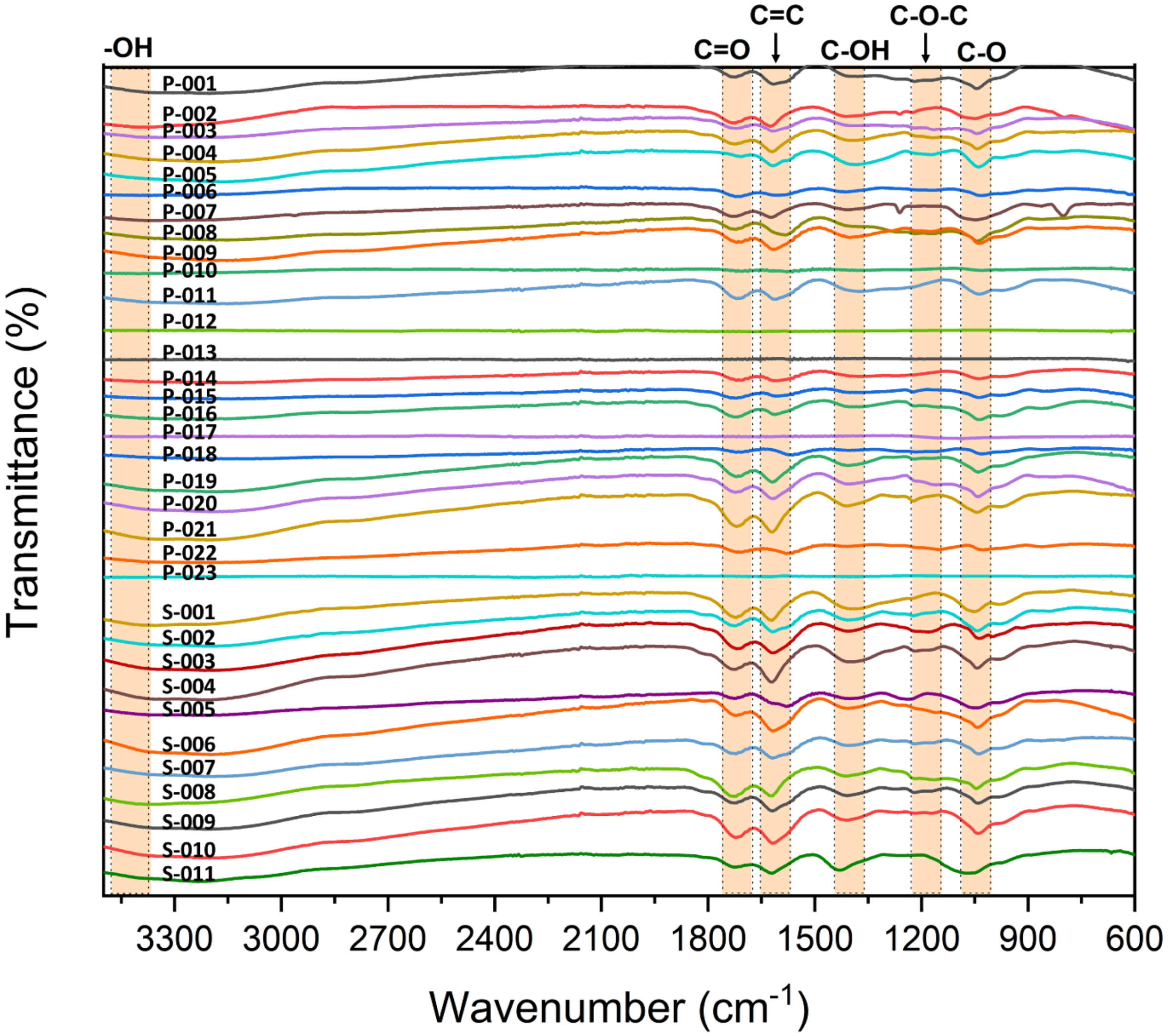

Figure S14. ATR spectra for all the samples.

Table S6. ATR data

Sample	Functional group	-OH	C=O	C=C	C-OH	C-O-C	C-O	GO-SO_3H*
	**Wavenumber (cm^{-1})**	3412-3420	1720-1740	1590-1627	1356-1365	1225-1260	1056-1060	1175, 1126, and 1040[16]
**P-001**		✓	✓	✓	✓	✓	✓	✓
**P-002**		✓	✓	✓	✓	✓	✓	-
**P-003**		✓	✓	✓	✓	✓	✓	✓
**P-004**		✓	✓	✓	✓	✓	✓	✓
**P-005**		✓	✓	✓	✓	✓	✓	✓
**P-006**		✓	✓	✓	✓	✓	✓	✓
**P-007**		✓	✓	✓	✓	✓	✓	-
**P-008**		✓	✓	✓	✓	✓	✓	✓
**P-009**		✓	✓	✓	✓	✓	✓	✓
**P-010**		✓	✓	✓	✓	✓	✓	-
**P-011**		✓	✓	✓	✓	✗	✓	-
**P-012**		✗	✗	✓	✗	✗	✗	-
**P-013**		✗	✗	✗	✗	✓	✓	-
**P-014**		✓	✓	✓	✓	✓	✓	-
**P-015**		✓	✓	✓	✓	✓	✓	-
**P-016**		✓	✓	✓	✓	✓	✓	-
**P-017**		✗	✗	✓	✗	✗	✓	-
**P-018**		✓	✓	✓	✓	✓	✓	✓
**P-019**		✓	✓	✓	✓	✓	✓	✓
**P-020**		✓	✓	✓	✓	✓	✓	✓
**P-021**		✓	✓	✓	✓	✓	✓	-
**P-022**		✓	✓	✓	✓	✓	✓	-
**P-023**		✓	✗	✓	✓	✗	✓	-
**S-001**		✓	✓	✓	✓	✓	✓	-
**S-002**		✓	✓	✓	✓	✓	✓	-
**S-003**		✓	✓	✓	✓	✓	✓	✓
**S-004**		✓	✓	✓	✓	✓	✓	✓
**S-005**		✓	✓	✓	✓	✓	✓	-
**S-006**		✓	✓	✓	✓	✓	✓	✓
**S-007**		✓	✓	✓	✓	✓	✓	✓
**S-008**		✓	✓	✓	✓	✓	✓	✓
**S-009**		✓	✓	✓	✓	✓	✓	✓
**S-010**		✓	✓	✓	✓	✓	✓	✓
**S-011**		✓	✓	✓	✓	✓	✓	-

*at least one of the bands was present in the spectrum.

Table S7. FTIR data.

Sample	Functional group	-OH	C=O	C=C	C-OH	C-O-C	C-O	$GO\text{-}SO_3H$*
	**Wavenumber (cm^{-1})**	3412-3420	1720-1740	1590-1627	1356-1365	1225-1260	1056-1060	1175, 1126, and 1040[16]
**P-010**		✓	✓	✓	✓	✓	✓	✓
**P-012**		✓	✗	✓	✗	✗	✓	-
**P-013**		✓	✗	✓	✓	✗	✓	-
**P-017**		✓	✗	✓	✗	✗	✓	-
**P-023**		✓	✓	✓	✗	✓	✗	✓

*at least one of the bands was present in the spectrum.

12. Inductively Coupled Plasma Optical Emission spectroscopy (ICP-OES)

Inductively Coupled Plasma-Optical Emission Spectrometer (ICP-OES) was performed, using a Perkin Elmer Avio 500, to analyze residual metals in all GO samples. In total, 17 metal species were analyzed (Al, As, Ba, Ca, Ce, Co, Cr, Cu, Fe, K, Mg, Mn, Na, Pt, Se, V, and Zn), while 15 were presented in Figure 2f and 2 of them (As and Ce) were below the detection limits of the method for all GOs. Solid samples (obtained in solid form or lyophilized) were digested with HNO_3/HCl (3:1) in microwave at 240 °C for 15 min and top up to 14 mL with H_2O. Precipitate was observed prior to analysis. The results are gathered in **Table S8**.

Table S8. Summary of ICP-OES results

Elemental concentration (ppm)																	
**Samples**	**As/Ce**	**Se**	**Co**	**Ba**	**V**	**Pt**	**Cu**	**Al**	**Cr**	**Zn**	**Ca**	**Mg**	**K**	**Fe**	**Na**	**Mn**	**Total**
**P-001**	-	-	-	1	-	8	-	52	2	4	63	9	675	37	19	6134	7004
**P-002**	-	-	-	2	-	-	-	23	2	54	59	13	1751	64	103	4082	6153
**P-003**	-	-	-	47	-	2	84	255	31	41	2880	273	647	509	713	6161	11643
**P-004**	-	-	-	-	-	9	-	-	51	9	159	53	1019	236	221	4608	6365
**P-005**	-	-	-	-	-	-	-	-	24	6	47	90	-	240	3172	19	3598
**P-006**	-	-	-	2	-	-	-	-	18	12	592	575	628	64	2923	356	5170
**P-007**	-	-	-	2	-	8	-	82	8	90	268	45	1261	140	76	3930	5910
**P-008**	-	7	-	1	-	26	-	40	5	9	221	29	677	29	79	9138	10261
**P-009**	-	15	-	4	-	11	-	-	-	4	79	10	1356	52	32	3850	5413
**P-010**	-	-	-	6	-	1	-	132	170	85	201	60	724	791	920	737	3827
**P-011**	-	-	-	-	-	-	-	-	22	3	25	1	51	201	-	104	407
**P-012**	-	-	-	6	2	-	-	-	17	-	47	6	-	62	47	12	199
**P-013**	-	-	-	5	6	-	-	78	252	-	71	13	126	524	950	167	2192
**P-014**	-	-	-	-	-	-	-	72	16	4	19	1	83	174	-	101	470
**P-015**	-	10	-	12	-	-	-	206	39	4	196	238	-	413	7	6	1131
**P-016**	-	-	-	3	-	-	-	270	7	5	323	296	-	216	35	10	1165
**P-017**	-	-	25	22	24	-	61	3584	753	21	1140	2344	581	2616	31596	209	42976

S-011	S-010	S-009	S-008	S-007	S-006	S-005	S-004	S-003	S-002	S-001	P-023	P-022	P-021	P-020	P-019	P-018
-	-	-	-	-	-	-	-	-	-	-	-	-	-	-	-	-
-	-	-	-	-	-	-	-	-	-	-	-	-	-	-	5	-
-	-	-	-	-	-	-	-	-	-	-	5	60	-	-	-	1
5	-	-	-	-	11	-	1	-	-	-	14	51	-	-	3	42
-	-	-	-	-	-	-	-	-	-	-	-	3	-	-	-	3
-	-	-	-	-	5	-	-	-	-	-	3	5	3	38	-	2
-	-	-	-	-	2	-	-	-	-	-	27	143	-	-	-	3
36	2	-	-	-	939	-	-	-	-	-	120	70	-	-	46	251
2	-	-	-	-	8	4	4	14	-	7	1088	44	2	21	8	10
7	-	-	-	-	100	2	6	-	-	1	18	104	11	3	23	26
7695	3	3	3	3	1997	40	138	1	1	12	1193	4498	36	33	1700	149
2062	1	-	-	-	723	4	33	-	-	2	888	837	6	7	328	53
307	-	-	-	18	170	-	1168	-	4	24	1164	3665	2533	1131	2704	3724
34	13	-	1	2	1522	173	52	56	-	23	1727	2979	15	59	156	294
1172	-	-	3	-	1212	1	163	-	-	6	1943	3339	237	71	185	49
5	3	21	0	253	9385	25	3222	1	30	7	2714	4068	1904	12570	756	1274
11325	22	24	7	276	16074	249	4787	72	35	82	10904	19866	4747	13933	5914	5881

13. Dispersions’ apparent stability

Dispersion stability in water is a crucial characteristic of GO materials. Each water dispersion (0.05 mg/mL) was placed in a 20 mL glass vial and sonicated (30 min, 10 °C, Elmasonic S60H ultrasonic bath, 37 Hz). Pictures were taken directly, 24 h, and 30 days after the sonication to evaluate the short- and long-term stability of the suspensions. In general, samples provided as suspensions were much easier to disperse and were more stable in water even after 30 days (**Figure S15**). Even though some precipitate was formed, it was not compact and could be easily resuspended by simply shaking. On the other hand, concerning the powder samples, 9 out of 23 samples (P-001, P-005, P-008, P-010, P-012, P-013, P-017, P-018, and P-023) did not form homogeneous suspensions after 30 min of ultrasound and precipitated during or very shortly after sonication indicating lower oxidation degree or high level of contamination. Due to the higher flake compaction drying process, only 30% of powder samples were stable after 30 days with none or negligible easy-to-resuspend precipitate (P-002, P-004, P-007, P-011, P-014, P-015, and P-021).

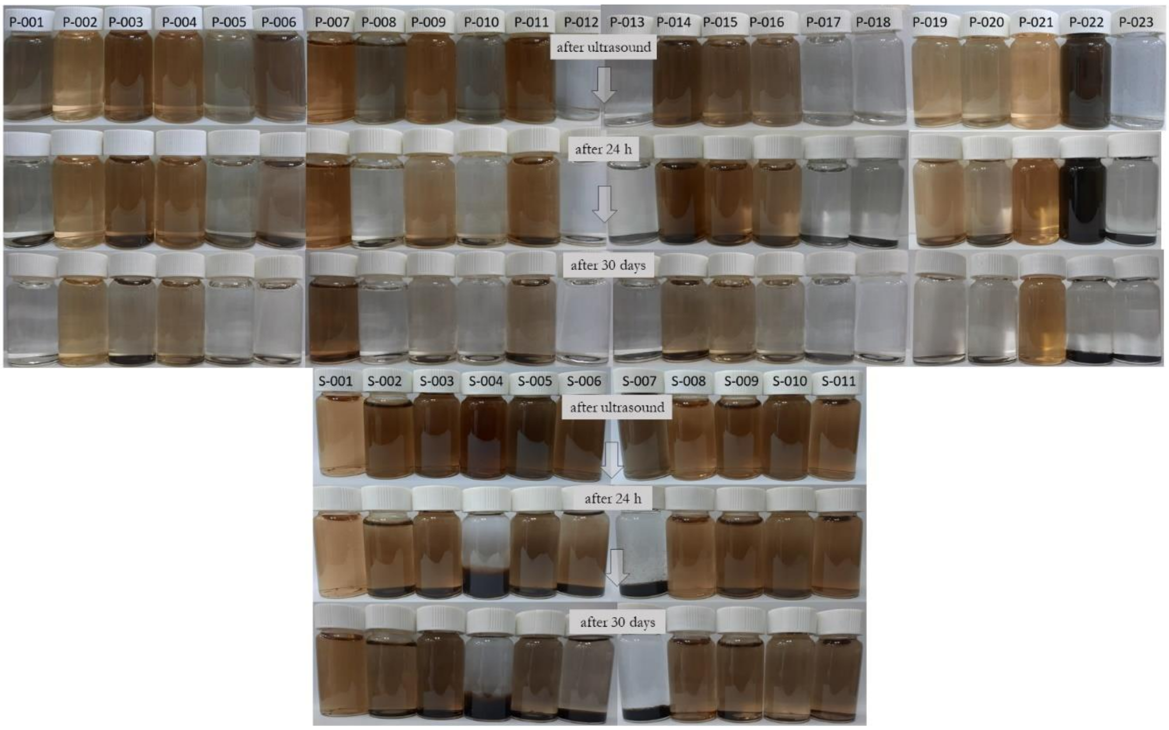

Figure S15. Dispersion stability in water directly after 30 min ultrasound, 24 hours and 30 days.

14. Gravimetric Study

All the GO suspensions/slurries were analyzed for the real concentration determination. This process involved the use of a freeze dryer to remove the water content from the suspension. For this, 30 mL of well homogenized GO suspensions were weighed, fully frozen, and loaded into the freeze dryer (Esco SubliMate®2, FDL-2S8). The lyophilization was performed at -40 °C, under the pressure of ~0.323 mbar, for a minimum of 48 h. Then, the samples were placed in the desiccator and left there until no significant mass change. All samples were lyophilized in triplicate (**Table S9**). Obtained solids were submitted for further analysis.

Table S9. Gravimetric analysis of GO suspension/slurries in triplicate and comparison with the concentration provided by the seller.

Sample	Displayed Concentration (mg/mL)	Real concentration (mg/mL)
**S-001**	5	4.1 ± 0.0
**S-002**	4	3.0 ± 0.5
**S-003**	1	0.6 ± 0.1
**S-004**	10	9.6 ± 0.3
**S-005**	2.5	2.1 ± 0.2
**S-006**	2	2.2 ± 0.1
**S-007**	8	8.2 ± 0.2
**S-008**	10	11.2 ± 0.0
**S-009**	2	2.3 ± 0.2
**S-010**	5	3.7 ± 0.3
**S-011**	20	3.6 ±0.4

Samples S-003, S-004, S-005, S-006, S-007, and S-009 matched (± 0.5 mg/mL) or exceeded (S-008) the seller concentration indication on the bottle. However, samples S-001, S-002, S-010, and especially S-011 demonstrated much lower real concentration (5x lower for S-011) than the one provided on the bottle. Obtained solids are present in **Figure S16.**

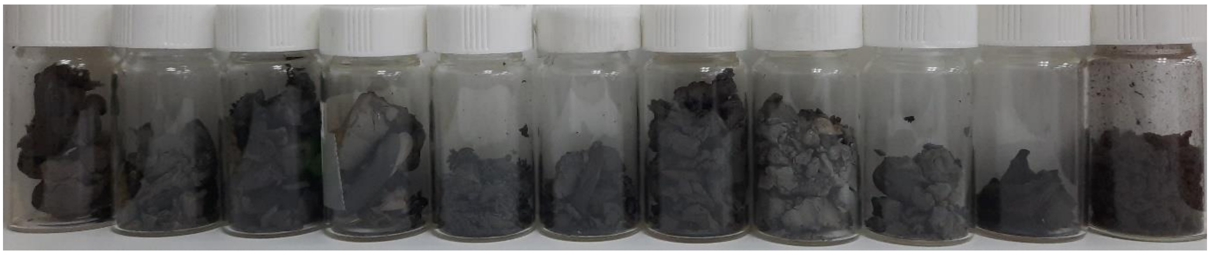

Figure S16. Lyophilized solids from left S-001 to S-011.

15. Ultraviolet–visible spectroscopy (UV-Vis)

GO dispersions (0.05 mg/mL) were sonicated using an ultrasound bath (Elmasonic S60H ultrasonic bath, 37 Hz) at 10 °C for 30 min. The samples were equilibrated to room temperature before the measurement. Double beam Lambda 750 UV–Vis spectrophotometer (Perkin Elmer, USA) was used to obtain the absorption spectra of GO. The GO samples were scanned for the wavelength range from 200 nm to 800 nm, with a 0.5 nm step, and a quartz cuvette with a 1 cm optical path. DI water was used as a blank to adjust the baseline. The measurement was repeated for 3 cycles per sample run and the average was calculated.

UV-Vis absorption measurements were conducted to explore the difference in the GO samples at the same concentration (0.05 mg/mL). GO has two characteristic absorption peaks in the UV-Vis spectra. The absorption peak at the region of 230 nm is assigned to $\pi \rightarrow \pi^*$ transitions of aromatic C=C bonds. Another characteristic feature, the shoulder peak at the region of 300 nm, is attributed to the n-π^* transition of C=O bonds[18]. Among the tested powder and suspension samples, there are nine samples (P-005, P-008, P-010, P-012, P-013, P-017, P-018, P-022, and P-023) that do not exhibit typical GO UV-Vis absorption spectra (**Figure S17a**). The absorption peak and shoulder peak wavelength of P-008 are shown to be out of the typical wavelength range, whereas P-010 does not have a defined shoulder peak at the 300 nm region. P-005 and P-022 show multiple peaks at the region of 230 nm. For the other five samples, the spectra appear as noisy spectrum signals without defined peaks.

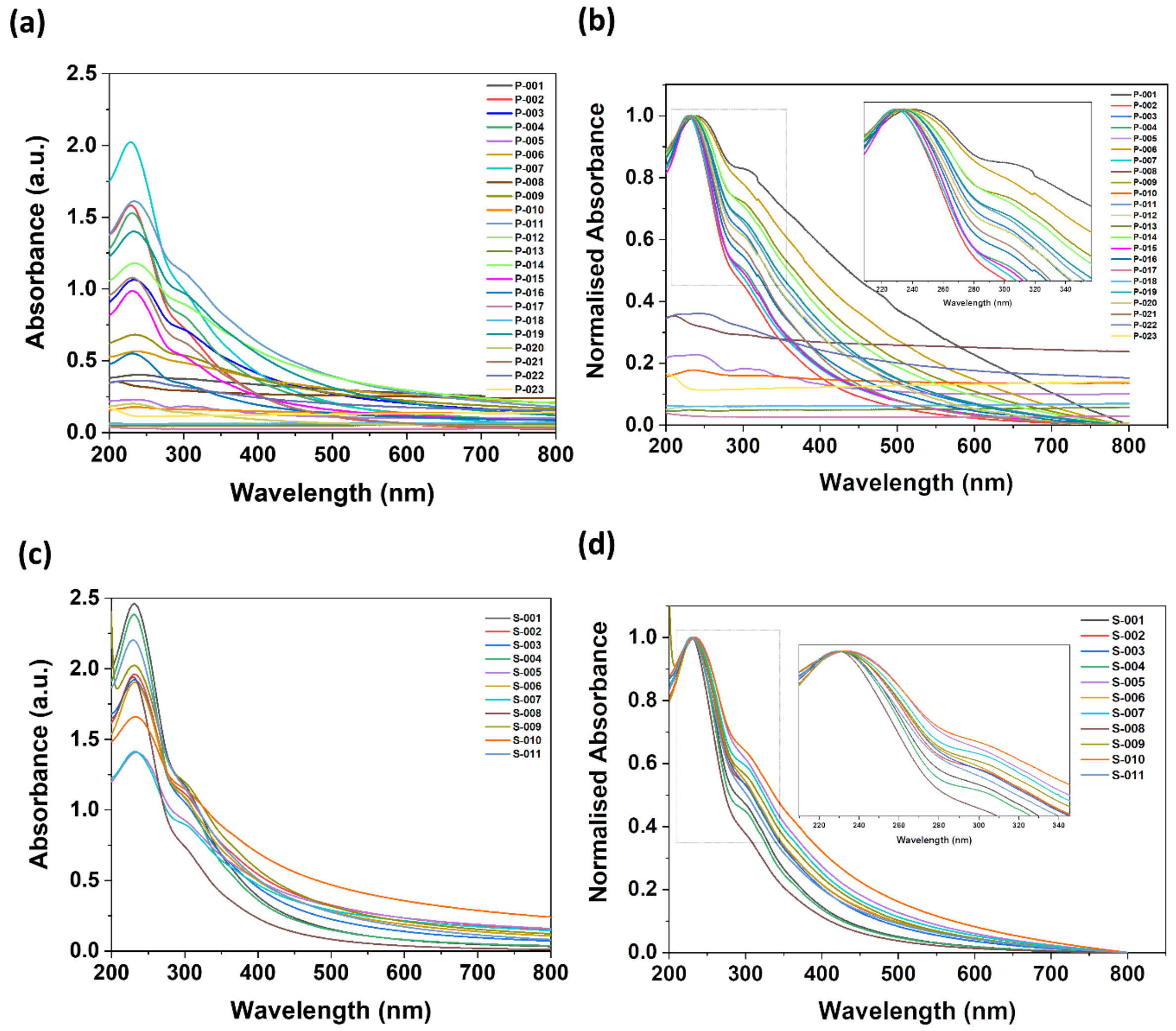

Figure S17. UV-vis absorption spectra and normalized spectra of GO aqueous dispersions from (a and b) powder samples and (c and d) suspension samples.

For the samples which exhibited typical characteristic absorption spectra, the intensity and position of each peak were shown to be different from each other. The degree of remaining conjugation in GO can be determined based on its λ_{max} of UV-Vis spectra. When there are more $\pi \rightarrow \pi^*$ transitions, less energy is needed for the electronic transition. Therefore, a higher λ_{max} will be obtained[17]. As what has been observed from the normalized spectra (**Figure S17a** and **b**), the absorption peaks of P-007 and S-008 appear at the lowest wavelength (228 nm) as compared to the rest, showing that these samples might be oxidized with more functional groups on the basal planes[19]. Besides, the degree of oxidation of GO could be reflected by the absorption band at around 300 nm[20].

Based on Beer-Lambert's law, the concentration of solutes is proportional to the absorbance of the solution[21]. Therefore, the dispersion stability of the GO dispersions can also be revealed via the three sequential cycles of measurement. **Figure 3** shows absorbance spectra of samples with different levels of stability. Moreover, 5 different groups of samples with diverging UV-Vis profiles were identified and were correlated with their water stability:

(1) Samples with very defined GO characteristic features (1 band and 1 shoulder), with negligible differences across the 3 repeated cycles and maximum absorbance between 1.4 and 2.5.
(2) Samples with defined GO characteristic features (1 band and 1 shoulder), but with small differences across the 3 repeated cycles and maximum absorbance between 0.7 and 2.5.
(3) Samples with defined GO characteristic features (1 band and 1 shoulder), but with more defined differences across the 3 repeated cycles (implying moderate stability) and maximum absorbance between 0.2 and 0.6.
(4) Samples with multiple bands and defined differences across the 3 repeated cycles (instability of dispersion), and with maximum absorbance between 0.16 and 0.375.
(5) Samples with multiple bands with very low absorbance values (below 0.2).

The most samples (group 1) are dominated by dispersion samples, with 9 GO dispersions (S-001, S-002, S-003, S-005, S-006, S-008, S-009, S-010, and S-011) and 3 GO powder samples (P-002, P-007, and P-011). The remaining 2 dispersion samples (S-004 and S-007) belong to group 2, together with other 7 powder samples (P-003, P-004, P-009, P-014, P-015, P-019, and P-021). All the remaining GOs are powder samples, where 9 of them present moderate to low stabilities and belong to group 3 (P-001, P-006, P-016 and P-020) and group 4 (P-005, P-008, P-010, and P-022). The lowest stabilities were observed for samples in group 5 (P-012, P-013, P-017, P-018, and P-023), where the UV-Vis spectra present very low intensities and the characteristic GO bands can barely be identified. Interestingly, the inferred UV-Vis stability groups closely match with the apparent long-term stability presented in **Figure S11**. The samples with very low stability (UV-Vis-based) are visually precipitated from the beginning (group 5), moderated to low stability ones were stable for more than 24 h (groups 3 and 4), and good stability samples were still stable after 30 days (groups 1 and 2).

16. pH and zeta potential measurements

It is known that pH of GO suspension impacts its degree of ionisation and aggregation while zeta potential (ζ) provides information about the effective surface charge of the interfacial double layer that indicates how stable the particle is dispersed in the medium[22]. In general, particles with ζ values of about ±30 mV would give a stable dispersion, and those with ±60 mV show excellent stability[23]. On the other hand, the closer the ζ values is to 0 mV, the higher the likelihood of aggregation[24]. At lower pH, the carboxyl groups on GO would be mostly protonated which destabilizes GO and cause aggregation. At a slightly higher pH and onwards, the carboxyl groups are mostly deprotonated and hydrophilic, and the GO sheets are dissolved in water, stabilized by the electrostatic repulsion from the various functional groups. GO has good dispersibility in water in pH range of 4 to 11.5[22]. Knowing how the pH effects the activity GO is essential in maximizing its applications. It is useful in tailoring GO for its different uses such as in films for electronics[22], formulation with salts for ionic sieving applications[25] and as dispersing agents[23,24].

pH and ζ measurements were performed simultaneously using Malvern Panalytical MPT-3 Autotitrator (with liquid-filled micro pH probe kit) and analyzed using Malvern Panalytical ZS XPLORER software. pH calibration was executed before running the first titration of the day. pH probe was calibrated using the Mettler Toledo InLab® Solution certified buffer solutions (pH 4.01, 7.00, and 9.21). 13 mL GO dispersions (0.05 mg/mL) were sonicated (30 min, 10 °C) and transferred into 25 mL-sample and titrant container (Malvern Panalytical, model no.: BEK 0008). The average results of the measurements at original and at adjusted (pH 6-7) pH are gathered in **Table S10** and **Figure S18.**

Table S10 Average ζ results of the measurements at original and at adjusted (pH 6-7) pH.

Sample	Original pH	ζ (mV)	ζ at pH 6-7 (mV)	ΔpH	Δ ζ (mV)
**P-001**	5.5±0.04	-42.1±1.6	-40.3±2.2	1.0	1.8
**P-002**	5.7±0.03	-52.2±2.3	-52.7±2.7	1.0	0.5
**P-003**	5.7±0.02	-46.7±0.7	-49.7±2.1	1.1	3.0
**P-004**	6.1±0.02	-42.7±3.5	-42.7±3.5	0.0	0.0
**P-006**	5.2±0.01	-49.9±2.6	-52.6±2.0	1.0	2.7
**P-007**	5.6±0.03	-53.9±1.4	-58.6±1.7	1.1	4.7
**P-009**	5.2±0.00	-48.1±3.4	-51.4±3.3	1.0	3.3
**P-011**	5.8±0.01	-45.0±0.8	-43.3±1.0	1.2	1.6
**P-014**	5.1±0.02	-36.3±1.6	-48.7±3.2	0.9	12.3
**P-015**	4.9±0.01	-41.3±2.4	-46.2±2.3	2.1	4.9
**P-016**	6.2±0.16	-52.9±4.7	-52.9±4.7	0.0	0.0
**P-019**	5.8±0.04	-47.2±1.6	-44.6±0.9	1.1	2.6
**P-020**	5.1±0.03	-48.6±5.0	-50.4±0.4	1.1	1.7
**P-021**	5.2±0.03	-50.1±2.9	-47.3±2.7	1.2	2.9
**S-001**	6.1±0.02	-58.7±1.8	-58.7±1.8	0.0	0.0
**S-002**	5.3±0.05	-48.2±2.1	-50.5±1.8	1.1	2.3
**S-003**	4.4±0.01	-43.4±3.6	-46.2±0.4	2.1	2.8
**S-004**	5.0±0.01	-56.9±4.6	-56.8±0.4	1.1	0.1
**S-005**	6.6±0.05	-51.0±3.4	-51.0±3.4	0.0	0.0
**S-006**	5.5±0.05	-45.5±1.1	-46.9±1.8	1.0	1.4
**S-007**	5.8±0.01	-48.7±2.1	-48.0±1.7	0.9	0.8
**S-008**	4.8±0.02	-51.5±0.7	-54.4±0.0	1.6	2.9
**S-009**	4.7±0.03	-45.3±1.8	-46.8±0.2	1.3	1.5
**S-010**	5.5±0.04	-51.6±0.5	-51.4±1.9	1.1	0.2
**S-011**	4.4±0.00	-38.2±1.0	-48.1±1.9	2.0	9.9

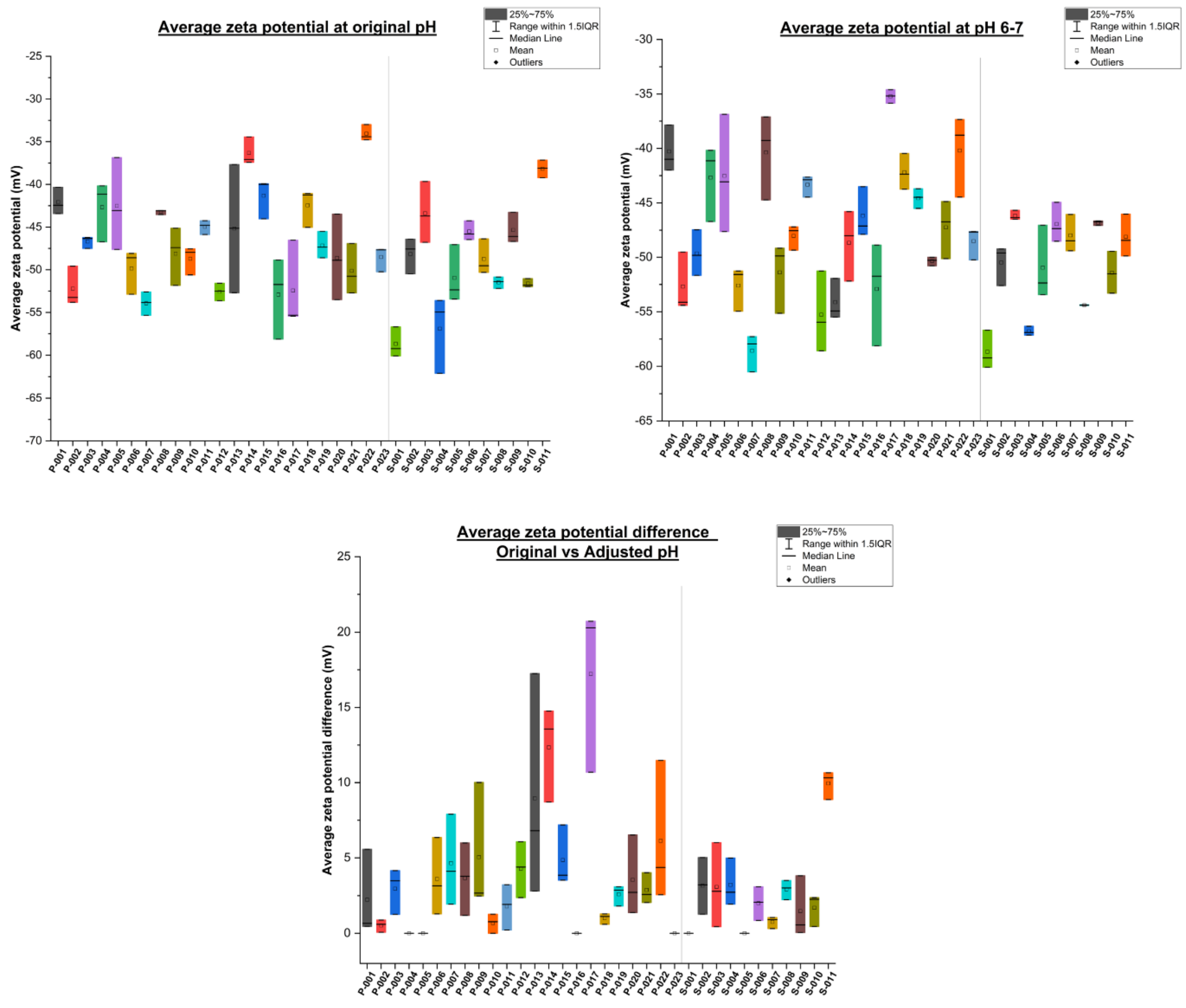

Figure S18. Comparison of ζ at original and adjusted pH.

The majority of samples presented original pH below 7 with the average value of 5.6. 50% of all GOs presented pH in the 5-6. The lowest pH of 4.4 was obtained for P-022, S-003, and S-011 while the highest pH of 8.6 was recorded for P-017. Additionally, samples P-012 and P-013 presented higher pH of 7.5 and 7.2, respectively.

Most of the samples presented ζ in the range (-)50-(-)45 mV at original (~35%) and adjusted pH (~32%), indicating good stability in water. The lowest value was obtained for sample GO-S-001 (-58.7±1.8 mV) for both original and adjusted pH. The highest values -34.1±0.9 (P-022) and -38.2±1.0 (S-011) for original pH and -35.2±0.6 mV (P-017) for adjusted pH.

17. Preparation of GO films and sheet resistivity measurements

The self-assembly ability of the GO samples into films and, subsequently, their measurements for sheet resistivity were performed with samples prepared at 1 mg/mL in water-ethanol (1:1) dispersion form. For GO samples in the aqueous suspension form, they were first diluted to 10 mL at 2 mg/mL with DI water and then with another 10 mL of ethanol (Fisher Chemical E/0650DF/17) to reach the 1 mg/mL dispersions. The dispersions were then ultrasonicated (Bandelin SONOREX) at 10 °C for 30 min to improve the exfoliation and homogeneity in the binary system. As for the GO samples in powder form or freeze-dried form, 10 mL of DI water was added to 20 mg of sample. The dispersion was first ultrasonicated for 15 min to ensure dissolution of the GO powder in water before ethanol was added, as GO solubility reduced with ethanol addition. Subsequently, another 10 mL of ethanol was added to reach the 1 mg/mL dispersion. Similar to the aqueous suspension samples, the 1 mg/mL dispersion was then ultrasonicated for another 30 min. The dispersion samples were then centrifuged (HETTICH ZENTRIFUGEN Tuttlingen D-78532) at 6000 rpm for 15 min at 22 ºC to separate the GO sheets by their size and net density[26,27].

In this isopycnic centrifugation step, the GO sheets that are thicker or with higher net density will precipitate. A higher net density sheet could occur from a lower degree of hydration that is caused by lesser functional groups found on the GO sheets. An observation that can be made from this step is that the better exfoliated and more functionalized GO samples tend to stay in the supernatant after centrifugation. The supernatants obtained from all the samples were then poured on polystyrene weighing boats and left to dry. **Figure S19** shows the products obtained after casting and solvent evaporation.

Samples that formed a film as indicated in **Figure S19** and **Table S11** were dried under vacuum for 2 h at 45 °C, followed by sheet resistivity measurements using the Four-Point Probe method for circular samples[28,29]. This is for comparing the sheet resistivities among the samples with the above-outlined preparation method and correlated with their previously displayed properties. Moreover, GO is well understood to be an insulator, this evaluation of resistivity can be used as a method to show the variation across the different GO samples.

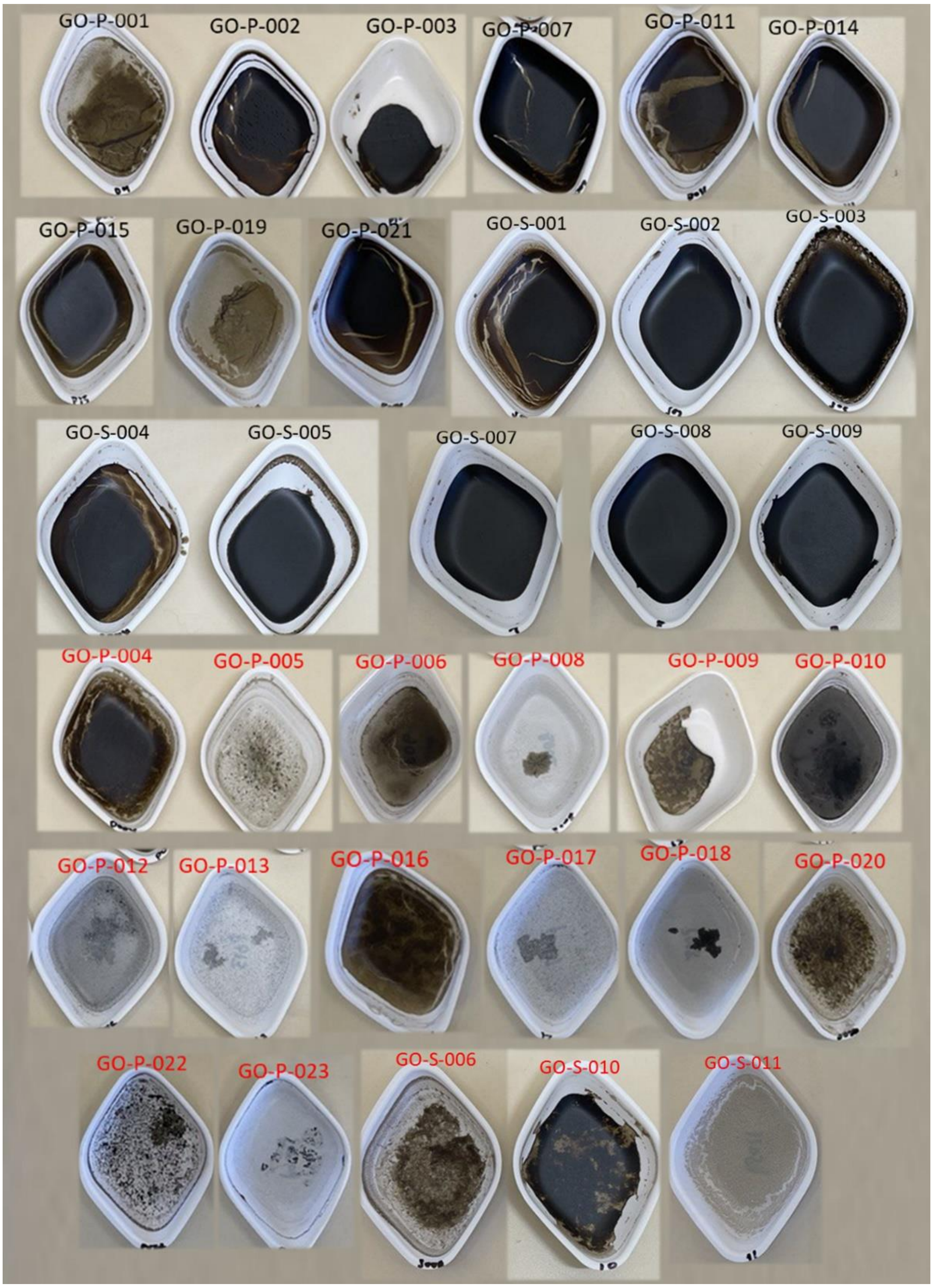

Figure S19 Photographic images of GO films formation attempt. Samples' names written in black means the system formed free standing film, while the ones written in red formed discontinuous structures that could not be characterized.

To note, the sheet resistivity value based on the Four-Point Probe measurement is valid without the geometric correction factor as long as the thickness of the input film is less

than 40% of the probe spacing, and the lateral size (diameter) is sufficiently larger than the distance between the probes. In our case, the film thicknesses were all below 100 μm (see Table S11) and the film diameter was set to be ~35 mm respectively. These values fulfilled the conditions stated above with a probe spacing of 1.591 mm[30]. The mean of 5 measurements and the standard deviation of the samples are tabulated in **Table S11**.

Table S11. Sheet resistivity of self-assembled GO films.

**Sample**	**Sheet Resistivity (MΩ/sq)**	**Applied Current (nA)[a]**	**Thickness (μm)**
**P-001**	3.98 ± 0.50	100	5
**P-002**	9.99 ± 2.22	100	26
**P-003**	1.27 ± 0.74	100	30
**P-007**	44.21 ± 9.17	10	55
**P-011**	157.12 ± 25.01	10	20
**P-014**	140.44 ± 16.26	10	36
**P-015**	0.85 ± 0.06	1000	54
**P-019**	85.44 ± 17.94	10	4
**P-021**	1.07 ± 0.14	1000	35
**S-001**	97.77 ± 13.85	10	61
**S-002**	22.51 ± 3.51	10	20
**S-003**	1.15 ± 0.20	1000	33
**S-004**	1.93 ± 0.59	1000	42
**S-005**	125.94 ± 17.61	10	34
**S-007**	98.76 ± 30.40	10	25
**S-008**	0.62 ± 0.11	1000	42
**S-009**	2.01 ± 0.90	1000	41

[a] Variation of applied current is used due to the instrument's limit of measuring ΔV, whereby currents that are too high or low are not able to give a sheet resistivity reading. It is to be noted that the current and resistivity are inversely proportionate, which was also observed in the measurements taken above, that samples that have higher sheet resistivity require a lower input current to obtain a reading.

[b] To note measurement upper limit of the setup is 1 GΩ/sq.

18. Individual GO samples profile

Table S12. Summary of the commercial GOs' individual structural and compositional features, and properties.

Sample	Morphology/Structure			Composition				Properties	
	Lateral size - AFM (µm)	Lateral size -SEM (µm)	Average Thickness (nm) (1)	O/C (2)	sp^3 (3)	I_D/I_G (4)	Total Residue (ppm)(5)	Stability in water (6)	Sheet resistivity(7)
**P-001**	0.886	0.2456	33.4	1.09	38.28	1.05	7004	3	3.98
**P-002**	4.730	1.1283	19.6	1.19	30.80	0.98	6153	1	9.99
**P-003**	1.282	0.1816	23.9	1.10	29.28	1.11	11643	2	1.27
**P-004**	2.337	0.2834	4.1	1.17	30.68	1.05	6365	2	Χ
**P-005**	1.168	0.3298	10.8	0.82	30.87	1.19	3598	4	Χ
**P-006**	0.872	0.2054	14.0	1.04	40.72	1.19	5170	3	Χ
**P-007**	1.389	0.3588	3.4	1.07	22.87	1.01	5910	1	44.21
**P-008**	1.952	0.4180	39.3	1.07	28.32	1.06	10261	4	Χ
**P-009**	0.580	0.2133	19.5	1.02	40.64	1.06	5413	2	Χ
**P-010**	1.034	Χ	93.3	0.49	35.74	1.05	3827	4	Χ
**P-011**	0.699	0.5316	2.9	0.85	26.14	1.04	407	1	157.12
**P-012**	Χ	Χ	Χ	0.03	0.00	0.33	199	5	Χ
**P-013**	Χ	Χ	Χ	0.05	12.25	0.45	2192	5	Χ
**P-014**	2.973	Χ	69.1	0.88	22.7	1.06	470	2	140.44
**P-015**	1.658	0.2310	38.0	0.92	36.96	1.01	1131	2	0.85
**P-016**	1.857	0.3190	18.3	0.93	33.62	1.04	1165	3	Χ
**P-017**	Χ	Χ	Χ	0.14	0.00	0.91	42976	5	Χ
**P-018**	0.958	Χ	197.2	0.76	34.08	0.78	5881	5	Χ
**P-019**	1.334	0.2977	13.6	0.97	29.92	1.07	5914	2	85.44
**P-020**	4.224	0.3987	21.9	1.03	31.03	1.07	13933	3	Χ
**P-021**	0.342	0.1648	1.6	1.08	53.87	1.02	4747	2	1.07
**P-022**	0.401	0.1811	10.2	1.09	41.95	1.09	19866	4	Χ
**P-023**	2.626	0.1822	569.0	0.11	30.65	1.06	10904	5	Χ
**S-001**	0.983	0.1468	16.7	0.83	25.68	1.04	82	1	97.77
**S-002**	0.411	0.4263	3.0	0.92	33.06	1.09	35	1	22.51
**S-003**	0.668	0.1935	1.8	1.02	34.99	1.11	72	1	1.15
**S-004**	0.403	0.2258	1.7	1.05	25.06	1.01	4787	2	1.93
**S-005**	1.049	0.2632	22.8	0.82	29.16	1.13	249	1	125.94
**S-006**	0.920	0.2097	3.7	1.03	39.43	1.07	16074	1	Χ
**S-007**	0.603	0.1765	11.2	0.97	34.56	1.04	276	2	98.76
**S-008**	0.332	0.4595	1.6	1.20	26.79	1.01	7	1	0.62
**S-009**	1.202	0.2185	15.4	1.04	35.71	1.07	24	1	2.01
**S-010**	1.203	0.1666	55.0	0.91	37.91	1.09	22	1	Χ
**S-011**	0.214	0.2977	1.7	1.11	30.87	1.03	11325	1	Χ

(1) Obtained by AFM. (2) Obtained by EA. (3) Obtained by XPS. (4) Obtained by Raman. (5) Obtained by ICP-OES. (6) Obtained by UV/Vis and following the stability groups presented in Fig. 3b. (7) Obtained by Four-Point Probe method.

References

1. Liscio, A. *et al.* Evolution of the size and shape of 2D nanosheets during ultrasonic fragmentation. *2D Mater.* **4**, 025017 (2017).

2. Jung, I., Rhyee, J. S., Son, J. Y., Ruoff, R. S. & Rhee, K. Y. Colors of graphene and graphene-oxide multilayers on various substrates. *Nanotechnology* **23**, 025708 (2012).

3. Backes, C. *et al.* Guidelines for exfoliation, characterization and processing of layered materials produced by liquid exfoliation. *Chem. Mater.* **29**, 243–255 (2017).

4. Park, J. *et al.* A study of the correlation between the oxidation degree and thickness of graphene oxides. *Carbon N. Y.* **189**, 579–585 (2022).

5. Li, Z. *et al.* Graphene Quantum Dots Doping of MoS2 Monolayers. *Adv. Mater.* **27**, 5235–5240 (2015).

6. ISO/TS 21356-1: Nanotechnologies — Structural characterization of graphene — Part 1: Graphene from powders and dispersions. vol. 2011 (2011).

7. Chen, J. *et al.* Size Fractionation of Graphene Oxide Sheets via Filtration through Track-Etched Membranes. *Adv. Mater.* **27**, 3654–3660 (2015).

8. Poulin, P. *et al.* Superflexibility of graphene oxide. *Proc. Natl. Acad. Sci. U. S. A.* **113**, 11088–11093 (2016).

9. Kime, G. *et al.* PH Dependence of Ultrafast Charge Dynamics in Graphene Oxide Dispersions. *J. Phys. Chem. C* **123**, 10677–10681 (2019).

10. Rodrigues, A. F. *et al.* A blueprint for the synthesis and characterisation of thin graphene oxide with controlled lateral dimensions for biomedicine. *2D Mater.* **5**, 035020 (2018).

11. Vacchi, I. A., Raya, J., Bianco, A. & Ménard-Moyon, C. Controlled derivatization of hydroxyl groups of graphene oxide in mild conditions. *2D Mater.* **5**, 035037 (2018).

12. Kovtun, A. *et al.* Accurate chemical analysis of oxygenated graphene-based materials using X-ray photoelectron spectroscopy. *Carbon N. Y.* **143**, 268–275 (2019).

13. Shahriary Leila & Athawale Anjali A. Graphene Oxide Synthesized by using Modified

Hummers Approach. *Int. J. Renew. Energy Environ. Eng.* **02**, 58–63 (2014).

14. Childres, I., Jauregui, L. A., Park, W., Caoa, H. & Chena, Y. P. Raman spectroscopy of graphene and related materials. in *New Developments in Photon and Materials Research* 403–418 (2013).

15. Farivar, F. *et al.* Unlocking thermogravimetric analysis (TGA) in the fight against "Fake graphene" materials. *Carbon N. Y.* **179**, 505–513 (2021).

16. Si, Y. & Samulski, E. T. Synthesis of water soluble graphene. *Nano Lett.* **8**, 1679–1682 (2008).

17. Marcano, D. C. *et al.* Improved Synthesis of Graphene Oxide. *ACS Nano* **4**, 4806–4814 (2010).

18. Paredes, J. I., Villar-Rodil, S., Martínez-Alonso, A. & Tascón, J. M. D. Graphene oxide dispersions in organic solvents. *Langmuir* **24**, 10560–10564 (2008).

19. Emiru, T. F. & Ayele, D. W. Controlled synthesis, characterization and reduction of graphene oxide: A convenient method for large scale production. *Egypt. J. Basic Appl. Sci.* **4**, 74–79 (2017).

20. Ahmad, M. A., Aslam, S., Mustafa, F. & Arshad, U. Synergistic antibacterial activity of surfactant free Ag–GO nanocomposites. *Sci. Rep.* **11**, 1–9 (2021).

21. Akhavan-Zanjani, H., Saffar-Avval, M., Mansourkiaei, M., Sharif, F. & Ahadi, M. Experimental investigation of laminar forced convective heat transfer of Graphene-water nanofluid inside a circular tube. *Int. J. Therm. Sci.* **100**, 316–323 (2016).

22. Shih, C.-J., Lin, S., Sharma, R., Strano, M. S. & Blankschtein, D. Understanding the pH-Dependent Behavior of Graphene Oxide Aqueous Solutions: A Comparative Experimental and Molecular Dynamics Simulation Study. *Langmuir* **28**, 235–241 (2012).

23. Kazi, S. N. *et al.* Investigation on the use of graphene oxide as novel surfactant to stabilize weakly charged graphene nanoplatelets. *Nanoscale Res. Lett.* **10**, 1–15 (2015).

24. Seong, H. J. *et al.* Experimental Study on Characteristics of Grinded Graphene Nanofluids with Surfactants. *Materials (Basel).* **11**, (2018).

25. Baskoro, F. *et al.* Graphene oxide-cation interaction: Inter-layer spacing and zeta potential changes in response to various salt solutions. *J. Memb. Sci.* **554**, 253–263 (2018).

26. Cotet, L. C. *et al.* Versatile self-assembled graphene oxide membranes obtained under ambient conditions by using a water–ethanol suspension. *J. Mater. Chem. A* **5**, 2132–2142 (2017).

27. Sun, X., Luo, D., Liu, J. & Evans, D. G. Monodisperse chemically modified graphene obtained by density gradient ultracentrifugal rate separation. *ACS Nano* **4**, 3381–3389 (2010).

28. Zrudsky, D. R., Bush, H. D. & Fassett, J. R. Four Point Sheet Resistivity Techniques. *Rev. Sci. Instrum.* **37**, 885 (2004).

29. Lord, A. M. *et al.* The 100th anniversary of the four-point probe technique: the role of probe geometries in isotropic and anisotropic systems. *J. Phys. Condens. Matter* **27**, 223201 (2015).

30. Ossila. Sheet Resistance Equations and Theory | Complete Guide | Ossila. (2021).
